# Targeting plasticity in the pyrimidine synthesis pathway potentiates macrophage-mediated phagocytosis in pancreatic cancer models

**DOI:** 10.1172/JCI193370

**Published:** 2025-11-17

**Authors:** Jie Zhao, Xinghao Li, Xinyu Li, Pengfei Ren, Yilan Wu, Hao Gong, Lijian Wu, Junran Huang, Saisai Wang, Ziwei Guo, Mo Chen, Zexian Zeng, Deng Pan

**Affiliations:** 1 Department of Basic Medical Sciences, State Key Laboratory of Molecular Oncology, Tsinghua University, Beijing, China.; 2 Tsinghua-Peking Joint Centre for Life Sciences and; 3 Center for Quantitative Biology, Academy for Advanced Interdisciplinary Studies, Peking University, Beijing, China.

**Keywords:** Immunology, Metabolism, Oncology, Cancer immunotherapy, Innate immunity, Macrophages

## Abstract

Macrophage-mediated phagocytosis plays a critical role in the elimination of cancer cells and shaping antitumor immunity. However, the tumor-intrinsic pathways that regulate cancer cell sensitivity to macrophage-mediated phagocytosis remain poorly defined. In this study, we performed a genome-wide CRISPR screen in murine pancreatic cancer cells cocultured with primary macrophages and identified that disruption of the tumor-intrinsic pyrimidine synthesis pathway enhances phagocytosis. Mechanistically, we discovered that macrophages inhibit the pyrimidine salvage pathway in tumor cells by upregulating *Upp1*-mediated uridine degradation through cytokines TNF-α and IL-1. This shift increased tumor cells’ reliance on de novo pyrimidine synthesis. As a result, tumor cells with impaired de novo pyrimidine synthesis showed depleted UMP and displayed enhanced exposure of phosphatidylserine (PtdSer), a major “eat-me” signal, thereby promoting macrophage-mediated phagocytosis. In multiple pancreatic cancer models, *Cad*-deficient tumors exhibited markedly reduced tumor burden with increased levels of phagocytosis by macrophages. Importantly, the *Cad*-mediated suppression of pancreatic cancer was dependent on TAMs and cytokines IL-1 and TNF-α. Pharmacological inhibition of DHODH, which blocks de novo pyrimidine synthesis, similarly decreased tumor burden with enhanced phagocytosis in pancreatic cancer models. These findings highlight the critical role of the tumor-intrinsic pyrimidine synthesis pathway in modulating macrophage-mediated antitumor immunity, with potential therapeutic implications.

## Abstract 2

We discovered a tumor–macrophage metabolic crosstalk where tumor pyrimidine synthesis shapes macrophage anti-tumor responses, revealing new therapeutic opportunities.

## Introduction

Pancreatic ductal adenocarcinoma (PDAC) ranks among the deadliest human malignancies. Despite the remarkable advances in immunotherapy for various cancers, its efficacy in patients with PDAC remains limited, largely due to the tumor’s low immunogenicity and its immunosuppressive microenvironment (1, 2). This presents a major challenge in identifying new therapeutic targets for PDAC immunotherapy. Recent studies have revealed that the PDAC tumor microenvironment is characterized by a dense matrix enriched with tumor-associated macrophages (TAMs), which potentiate pathogenic inflammation in pancreatic cancer (2, 3), highlighting the potential of leveraging the antitumor effect of macrophages to treat PDAC. The dynamics of macrophage-mediated phagocytosis, particularly through interactions involving “eat-me” and “do-not-eat-me” signals, are essential in the recognition and elimination of cancer cells (3, 4). Eat-me signals could involve specific patterns of early apoptotic cells, such as phosphatidylserine (PtdSer), which could bind to receptors such as TIM4, on macrophages and trigger phagocytosis (5, 6). Cancer cells frequently express do-not-eat-me signals such as CD47, which binds to the inhibitory receptor SIRPα on macrophages, effectively blocking macrophage-mediated phagocytosis (7). The development of monoclonal antibodies targeting CD47 has shown encouraging results in enhancing antitumor immunity, highlighting the potential of these innate immune checkpoints as targets for cancer immunotherapy (8, 9). In addition to CD47, other regulators of phagocytosis in tumor cells, operating via distinct mechanisms, have been identified. For example, CD24 serves as a do-not-eat-me signal by interacting with Siglec-10 on macrophages, thereby inhibiting their phagocytic activity (10). MHC-I molecules interact with LILRB1/2 inhibitory receptors, aiding in the evasion of phagocytosis (11). Recently, genome-wide CRISPR screening has uncovered APMAP as a vital regulator that impedes macrophage-mediated phagocytosis by influencing the G protein–coupled receptor GPR84, a novel phagocytosis regulator expressed on macrophages (12). Despite these advances in understanding tumor-macrophage cell-cell interactions, the extent to which tumor cell–intrinsic pathways may interact with macrophages and subsequently interfere with the phagocytosis process remains largely unclear. Pyrimidine synthesis is essential for cellular growth and function, with de novo synthesis and salvage pathways as the two primary metabolic routes (13). The de novo pathway is initiated by the enzyme CAD (carbamoyl-phosphate synthetase II-aspartate transcarbamoylase-dihydroorotase), which catalyzes the formation of carbamoyl phosphate, and continues through DHODH (dihydroorotate dehydrogenase) and UMPS (uridine monophosphate synthetase) to generate UMP (uridine 5′-monophosphate), a precursor for nucleotide synthesis. The salvage pathway, on the other hand, recycles pyrimidine bases and nucleosides, converting uridine to UMP through UCK2 (uridine-cytidine kinase 2) and CDA (cytidine deaminase). Additionally, UMP metabolism can be regulated by UPP1 (uridine phosphorylase 1), which catalyzes the degradation of uridine to ribose-1-phosphate, balancing uridine availability (14). Dysregulation of pyrimidine metabolism plays a pivotal role in various types of cancer. For example, studies have shown that inhibition of DHODH leads to effective growth suppression in certain types of cancers, including isocitrate dehydrogenase mutant glioma, diffuse midline glioma, and a KRAS-driven PDAC model (15–17). Furthermore, CDA overexpression has been shown to elevate UDP levels, which modulate TAMs, inducing immune changes in the tumor microenvironment and promoting resistance to anti–PD-1 therapy (18). Altogether, these findings indicate that tumor pyrimidine metabolism could serve as an appealing therapeutic target.

In this study, we established an in vitro coculture system to model macrophage-tumor phagocytosis and utilized genome-wide CRISPR screening to identify potentially novel tumor-intrinsic genes and pathways regulating this process. Our findings revealed what we believe to be a novel relationship between macrophage-mediated phagocytosis and the tumor-intrinsic pyrimidine synthesis pathway operated by *Cad*, *Dhodh*, and *Umps*. We observed an enhanced phagocytic activity in macrophages when cocultured with tumor cells where the de novo pyrimidine synthesis pathway was inactivated. Mechanistically, we showed that proinflammatory cytokines released by macrophages, in particular TNF-α and IL-1, synergistically upregulate the expression of UPP1 in tumor cells. The upregulated UPP1, which catalyzes the degradation of uridine to uracil and ribose-1-phosphate, counteracts UMP synthesis, making the tumor cells more dependent on de novo synthesis of UMP. In the presence of macrophages releasing TNF-α and IL-1, tumor cells with a deficiency in de novo pyrimidine synthesis showed enhanced levels of PtdSer, a major eat-me signal, potentiating macrophage-mediated elimination in vitro and in mouse models. Thus, this study reveals a cytokine-metabolism crosstalk between macrophages and tumor cells.

## Results

### Loss of function of tumor-intrinsic de novo pyrimidine synthesis pathway sensitized pancreatic cancer cells to macrophage-mediated phagocytosis.

To pinpoint genes that modulate the vulnerability of tumor cells to macrophage-mediated phagocytosis, we devised a robust high-throughput screening assay. We genetically modified the Panc02 pancreatic cancer cells to express IgG Fc fragment in a reversed orientation on the cell surface (19), thereby simulating antibody-dependent cellular phagocytosis (ADCP) processes (Supplemental Figure 1A; supplemental material available online with this article; https://doi.org/10.1172/JCI193370DS1). We observed that Panc02 cells expressing the inverted Fc domain (Panc02-Fc; ZsGreen^+^) underwent substantial phagocytosis by bone marrow–derived macrophages (BMDMs, as indicated by the increased presence of ZsGreen and F4/80 double-positive cells)as compared with the vector control group (Supplemental Figure 1, B and C). As expected, the enhanced phagocytosis events were diminished in the presence of LAT-A, an inhibitor of actin polymerization and phagocytosis (20) (Supplemental Figure 1, B and C). To validate whether our system could identify tumor-intrinsic modulators of phagocytosis, we knocked out the expression of CD47, a well-established do-not-eat-me signal (7), in Panc02-Fc cells. While we observed only a modest depletion of CD47-KO cells in the presence of BMDMs, the depletion signal was notably more pronounced when IgG-Fc fragment was introduced (Supplemental Figure 1, D and E). Collectively, these results demonstrate that our coculture system imposes a strong selective pressure through macrophage-mediated phagocytosis on tumor cells.

**Figure 1:**
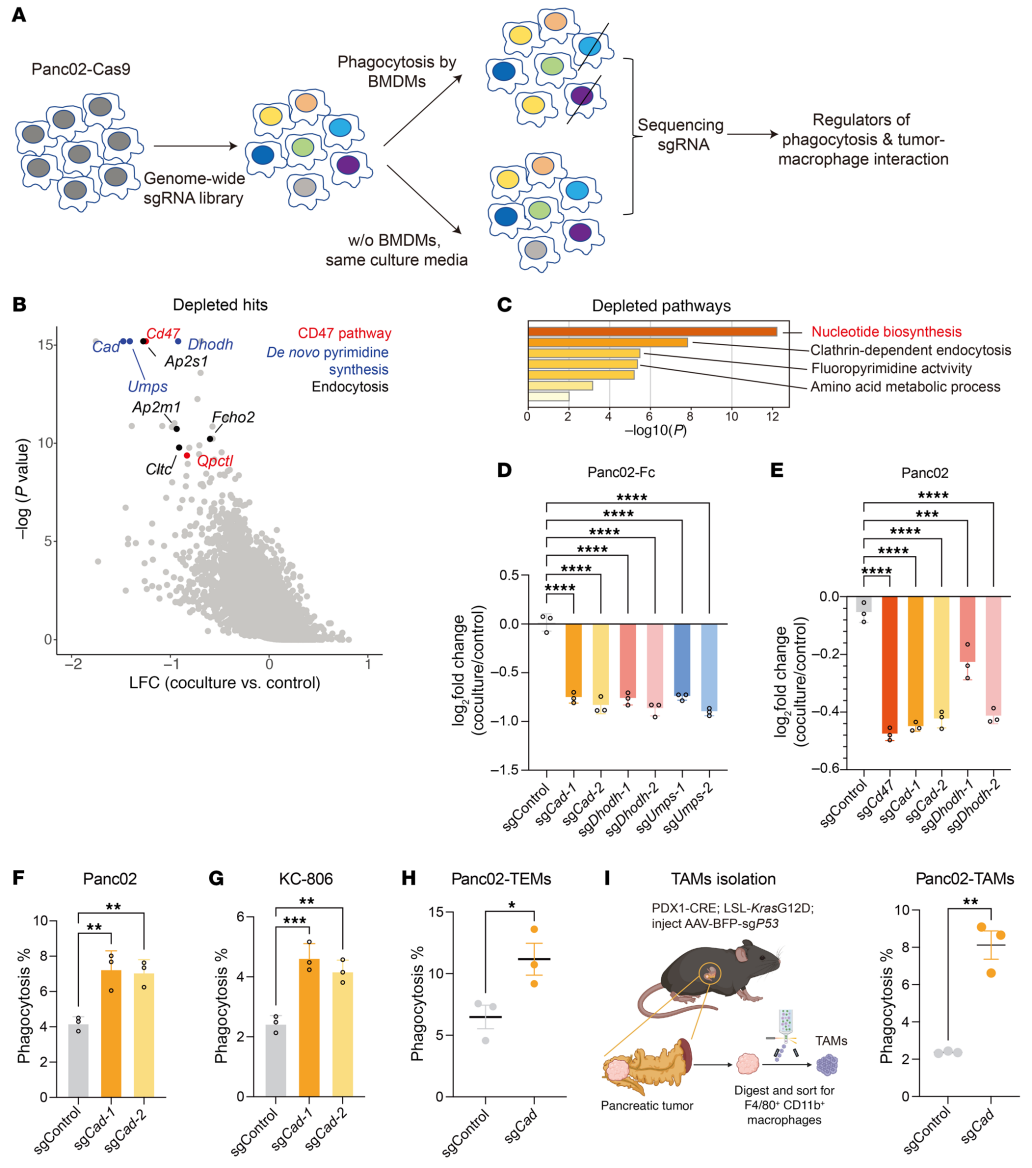
Loss of function of de novo pyrimidine synthesis pathway sensitized pancreatic tumor cells to macrophage-mediated phagocytosis. (**A**) Workflow of CRISPR screen for tumor-intrinsic regulators of macrophage-mediated phagocytosis. (**B**) Scatter plot showing the top depleted sgRNAs based on mean log_2_ fold change of sgRNA counts in BMDM coculture condition versus control condition. Annotated genes represent the CD47 pathway (red), the de novo pyrimidine synthesis pathway (blue), and endocytosis regulators (black). (**C**) Metascape analysis of pathways among top depleted hits (FDR < 0.1). (**D** and **E**) In vitro competition assay based on coculture of BMDMs and Panc02 tumor cells expressing Fc fragment (**D**) or parental Panc02 cells (**E**). Control Panc02-Fc cells were mixed with cells with indicated genes knocked out (labeled with CFSE) and then cocultured with BMDMs for 24 hours. Log_2_ fold change of the percentage of KO cells upon coculture with BMDMs was shown. (**F** and **G**) In vitro phagocytosis assay. BMDMs were cocultured with CFSE-labeled control or *Cad*-KO Panc02 (**F**) or KC-806 (**G**) cells for 24 hours. Percentages of phagocytosis (F4/80^+^ CSFE^+^) were quantified by FACS. (**H**) In vitro phagocytosis assay by using tumor-educated macrophages (TEMs). TEMs were cocultured with CFSE-labeled control or *Cad*-KO Panc02 cells for 24 hours. Percentages of phagocytosis (F4/80^+^CSFE^+^) were quantified by FACS. (**I**) In vitro phagocytosis assay by using TAMs isolated from an autochthonous pancreatic cancer model driven by *Kras*^G12D^ expression and *P53* inactivation. Coculture assays were performed using TAMs and CFSE-labeled Panc02 cells. Phagocytosis percentages (F4/80^+^CFSE^+^) were quantified by FACS. A schematic representation of TAM isolation from the autochthonous pancreatic tumor (created using BioRender.com) and statistical analysis are shown. Data are presented as mean ± SD and analyzed by 1-way ANOVA (**D**–**G**) or unpaired *t* test (**H** and **I**). *P < 0.05, **P < 0.01, ***P < 0.001, ****P < 0.0001. Data are representative of at least 2 independent experiments (**D**–**I**).

To uncover the intrinsic regulators of macrophage-mediated phagocytosis in cancer, we conducted a genome-wide CRISPR screen in Panc02-Fc cells, which were cocultured with BMDMs and then subjected to phagocytosis (Figure 1A). Notably, our screen revealed several top hits that regulate sensitivity to phagocytosis (Figure 1B and Supplemental Figure 2A). These top depleted hits include genes encoding the well-known antiphagocytic molecule CD47, the CD47-modifying enzyme QPCTL (21), suggesting that our screen could successfully recapitulate known regulators in the phagocytosis process (Figure 1B). Remarkably, sgRNAs targeting key enzymes in the de novo pyrimidine synthesis pathway (*Cad*, *Dhodh*, and *Umps*) were strongly depleted (Figure 1B), suggesting that inhibiting the de novo pyrimidine synthesis pathway might increase the susceptibility of Panc02 cells to macrophage-mediated phagocytosis. Consistently, the nucleotide biosynthesis pathway was the top enriched pathway among the depleted hits from the screen (Figure 1C). By comparing the changes in sgRNA abundance under normal cell culture conditions, we found that the inactivation of *Cad*, *Dhodh*, and *Umps* had negligible effects on tumor growth (Supplemental Figure 2B), ruling out the possibility that the loss of function of de novo pyrimidine synthesis leads to cell death through a nonspecific mechanism.

To confirm the role of pyrimidine synthesis enzymes in macrophage-mediated phagocytosis, we employed CRISPR/Cas9 to KO *Cad*, *Dhodh*, and *Umps* in Panc02-Fc cells (Supplemental Figure 2, C–E). Our in vitro competition assay revealed that KO of these genes substantially enhanced the susceptibility of Panc02-Fc cells to macrophage-mediated phagocytosis compared with the control group (Figure 1D). To determine whether these effects were specific to Fc-receptor–mediated phagocytosis, we replicated these KOs in both the parental Panc02 and the KC-806 cell line, the latter derived from the KC (*Kras*^G12D^; *Cdkn2a* KO) pancreatic cancer model, in which spontaneous pancreatic tumors were driven by oncogenic *Kras*^G12D^ and inactivation of *Cdkn2a* and *Smad4* (22). In both cell lines without Fc fragments, the KO of genes related to the de novo pyrimidine synthesis pathway similarly resulted in reduced cell numbers upon coculturing with BMDMs (Figure 1E and Supplemental Figure 2, F and G). Consistently, we observed an increased sensitivity to macrophage-mediated phagocytosis (Figure 1, F and G, and Supplemental Figure 2H). To investigate whether tumor cells become more susceptible to phagocytosis mediated by TAMs, we first cocultured *Cad*-KO Panc02 cells with tumor-educated macrophages (TEMs) generated by incubating BMDMs with tumor-derived supernatant. *Cad*-KO Panc02 cells were consistently more susceptible to TEM-mediated phagocytosis as compared with control cells (Figure 1H). To further validate these findings, we cocultured *Cad*-KO Panc02 cells with TAMs isolated from an autochthonous pancreatic cancer model (Figure 1I and Supplemental Figure 2I). This model was generated by injecting adeno-associated virus (AAV) carrying sgRNAs targeting the tumor suppressor gene p53 into LSL-Kras^G12D^; LSL-Cas9; Pdx1-Cre transgenic mice (23). Consistent with the TEM coculture experiments, *Cad*-KO Panc02 cells showed substantially increased susceptibility to TAM-mediated phagocytosis (Figure 1I). Collectively, these results indicate that *Cad* inactivation enhances the susceptibility of tumor cells to phagocytosis mediated by both TEMs and TAMs.

### Inactivation of tumor-intrinsic de novo pyrimidine synthesis pathway enhances macrophage-mediated antitumor effect in vivo.

**Figure 2:**
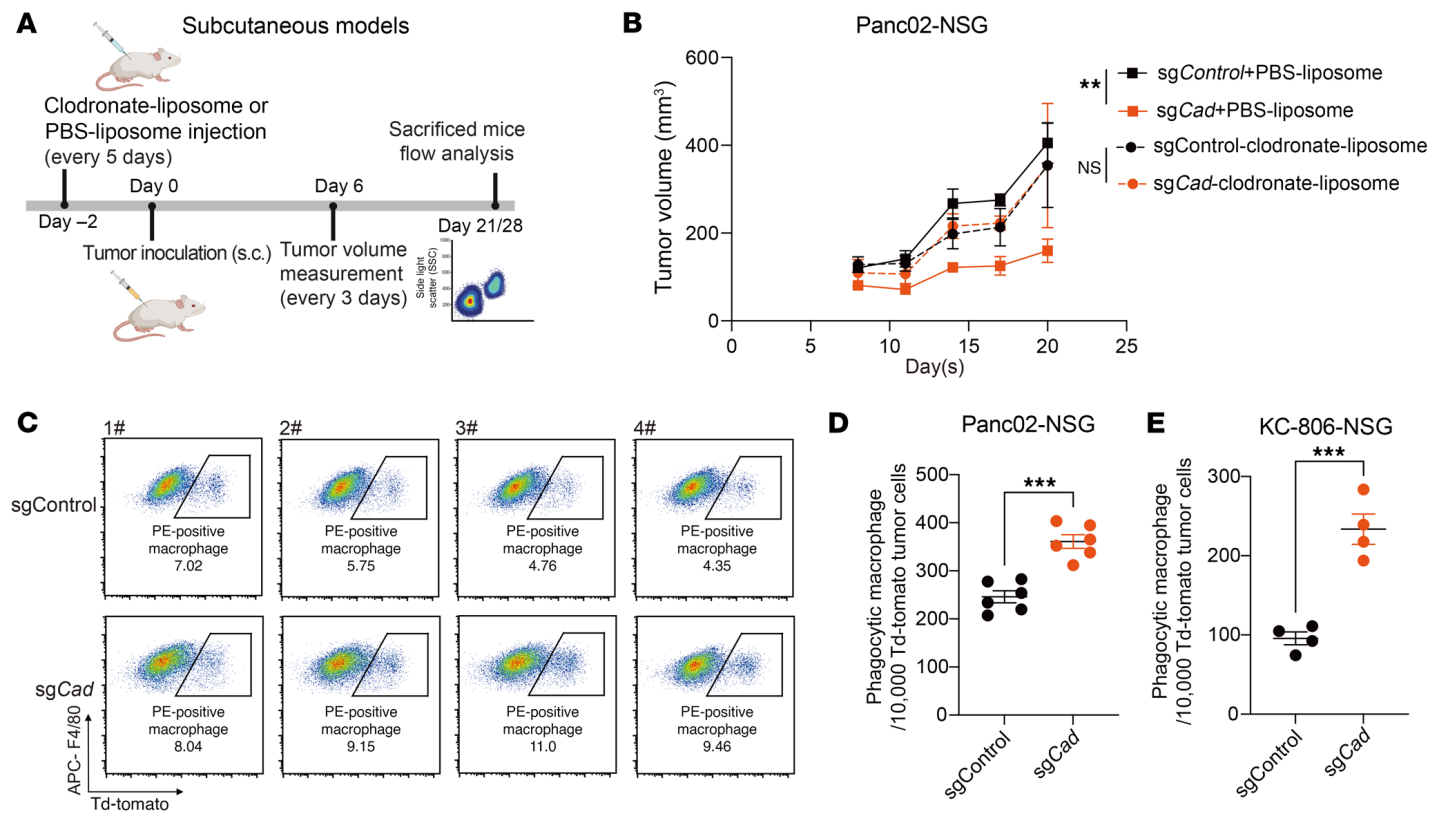
Inactivation of *Cad* potentiates macrophage-mediated tumor control and phagocytosis in subcutaneous models. (**A**) Schematic of pancreatic tumor subcutaneous administration. Created using BioRender.com. (**B**) Growth curves of control or *Cad*-KO Panc02 tumors following treatment of control vehicle (PBS liposome) or clodronate liposome in NSG mice. (**C**) Representative FACS plots of phagocytic macrophages in Panc02-Td-Tomato tumors. TAMs were gated on F4/80^+^ and Cd11b^+^. Phagocytic macrophages were identified as double positive for F4/80 and Td-Tomato. (**D** and **E**) Quantification of normalized cell number of phagocytic macrophages in Panc02-Td-Tomato (**D**) or KC-806-Td-Tomato (**E**) subcutaneous tumors. Data are represented as mean ± SEM and were analyzed by mixed-effects model (REML) test (**B**) or unpaired *t* test (**D** and **E**). **P < 0.01, ***P < 0.001. All data are representative of at least 2 independent experiments.

To explore the role of the tumor-intrinsic de novo pyrimidine synthesis pathway in modulating antitumor immunity in vivo, we first examined the effect of *Cad* KO in tumor growth using Panc02 tumor models. KO of *Cad* led to substantially slower tumor growth compared with control groups (Supplemental Figure 3A). To assess the effect of TAMs on *Cad*-deficient tumors, we depleted macrophages using clodronate liposomes prior to inoculating *Cad*-KO and control tumors into NSG and WT mice, respectively (Figure 2A and Supplemental Figure 3B). While *Cad* KO resulted in substantially slower tumor growth in both WT C57BL/6 (B6) and NSG mice, TAMs depletion by clodronate liposomes rescued the antitumor effects of *Cad* KO in both tumor models (Figure 2B and Supplemental Figure 3, C–E), indicating that the antitumor activity upon *Cad* inactivation relies on the presence of macrophages. Consistent with in vitro findings, the inactivation of *Cad* led to a substantial increase in phagocytosis levels, as demonstrated by the higher ratio of Td-Tomato^+^ macrophages (Cd11b^+^F4/80^+^) in both Panc02 and KC-806 mouse models (Figure 2, C–E, and Supplemental Figure 3, F–I). Taken together, these results suggest that tumors deficient in *Cad* enhanced macrophage-mediated antitumor effect and phagocytosis in vivo.

**Figure 3:**
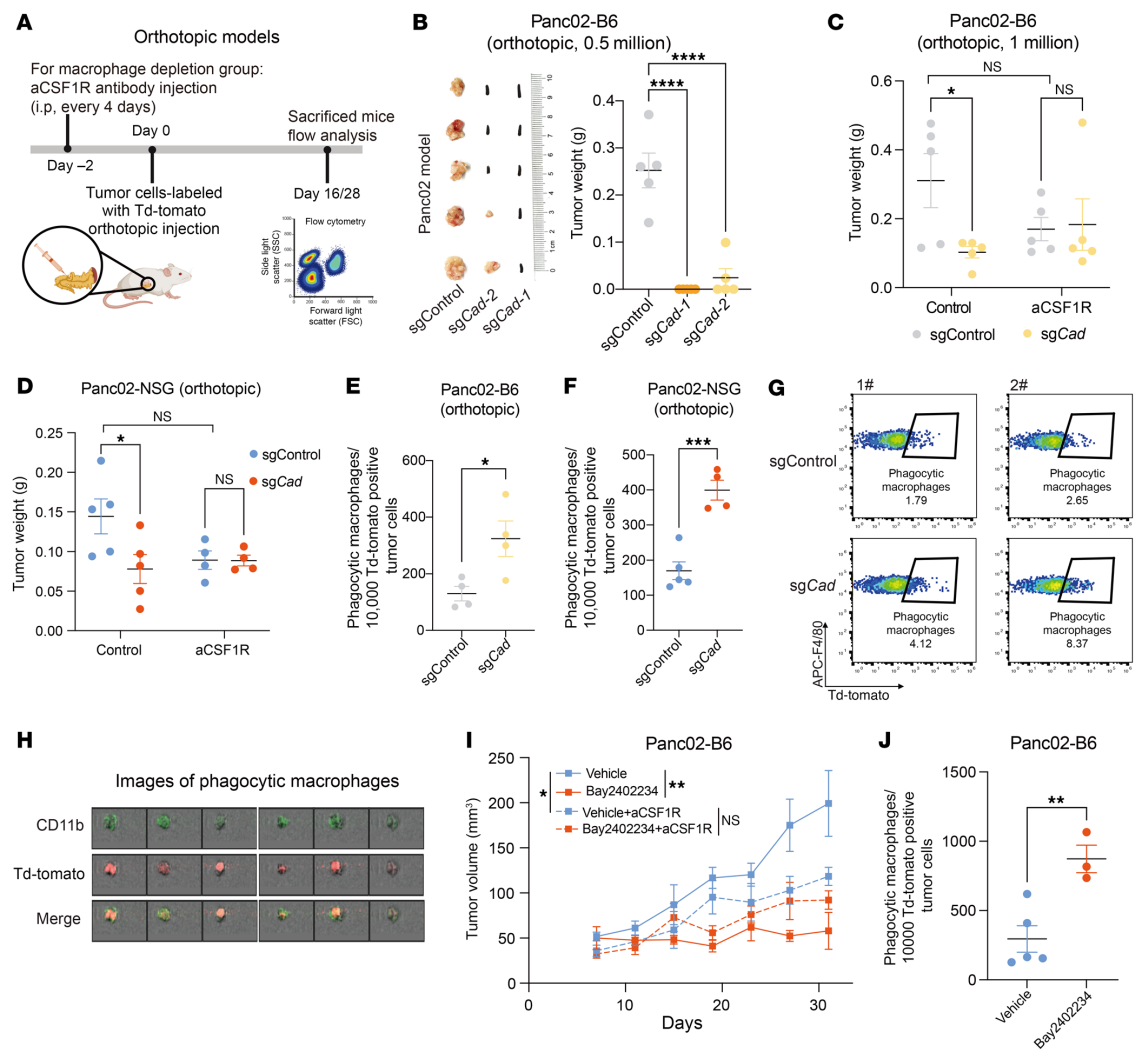
Inactivation of de novo pyrimidine synthesis pathway potentiates macrophage-mediated tumor control and phagocytosis in vivo. (**A**) Schematic of pancreatic tumor orthotopic administration. Created using BioRender.com. (**B**) 5 × 10^5^ of control or *Cad*-KO Panc02 cells were orthotopically implanted into B6 mice, and tumor weight was measured on day 28 after implantation. Tumor images (left) and weights (right) are shown. (**C**) 1 × 10^6^ of control or *Cad*-KO Panc02 cells were orthotopically implanted into B6 mice, with or without aCSF1R antibody treatment. Tumor weight was measured on day 28 after implantation. (**D**) 5 × 10^5^ of control or *Cad* KO-Panc02 cells was orthotopically implanted into NSG mice, with or without aCSF1R antibody treatment. Tumor weight was measured on day 16 after implantation. (**E**–**H**) Quantification of the normalized percentage of phagocytic macrophages in Panc02-Td-Tomato orthotopic tumors in B6 (**E**) and NSG (**F**) mice. Representative FACS plots are shown in **G**. Representative images showing overlay signals of CD11b (green) and Td-Tomato (red), as determined by imaging flow cytometry, are displayed in **H**. (**I** and **J**) B6 mice were inoculated with Td-Tomato-labeled Panc02 cells, treated with BAY2402234 or vehicle at 4mg/kg every 3 days (intratumorally), with or without anti-CSF1R antibody as indicated. Tumor growth curves (**I**) and statistics of phagocytic macrophages (**J**) are shown. Data are represented as mean ± SEM and analyzed by 1-way ANOVA (**B**), 2-way ANOVA (**C** and **D**), unpaired *t* test (**E**, **F**, and **J**), or mixed-effects model (REML) test (**I**). *P < 0.05, **P < 0.01, ***P < 0.001 and ****P < 0.0001. All data are representative of at least 2 independent experiments.

To better evaluate the relevance of our findings in pancreatic cancer, we orthotopically inoculated *Cad*-KO and control Panc02 cells into the pancreas of WT B6 or NSG mice, both of which contained TAMs (Figure 3A). Consistent with subcutaneous tumor models, *Cad* KO substantially reduced tumor burden in both B6 and NSG mice (Figure 3, B–D). Depletion of TAMs using aCSF1R antibodies (Supplemental Figure 3, J and K) resulted in comparable tumor burden between control and *Cad* KO tumors, indicating that macrophages are crucial for controlling orthotopically inoculated *Cad*-KO Panc02 tumors (Figure 3, B–D, and Supplemental Figure 3L). Additionally, *Cad* inactivation led to a higher proportion of phagocytic macrophages (Figure 3, E–H). We also validated these findings using the KC-806 tumor model orthotopically, obtaining consistent results that inactivation of *Cad* induced smaller tumors in a macrophage-dependent manner (Supplemental Figure 3, L and M) and enhanced phagocytosis (Supplemental Figure 3N). Together, these data indicate the inactivation of *Cad* potentiates macrophage-mediated tumor control in multiple pancreatic tumor models.

Next, we tested whether inhibition of de novo pyrimidine synthesis pathway could potentiate macrophage-mediated antitumor immunity. To this end, we administrated DHODH-specific inhibitor BAY2402234 to Panc02 tumors. Treatment of BAY2402234 substantially inhibited the growth of Panc02 tumor (Figure 3I and Supplemental Figure 3O). However, this DHODH inhibition–mediated antitumor effect was largely abolished when macrophages were depleted (Figure 3I and Supplemental Figure 3O). Consistently, we observed an increase of phagocytic macrophages in BAY2402234 treatment group compared with vehicle group (Figure 3J and Supplemental Figure 3P), indicating that pharmacological inhibition of DHODH could inhibit tumor growth and potentiate phagocytosis in pancreatic cancer model.

### Inactivation of Cad increased the exposure of PtdSer in the presence of macrophages.

**Figure 4:**
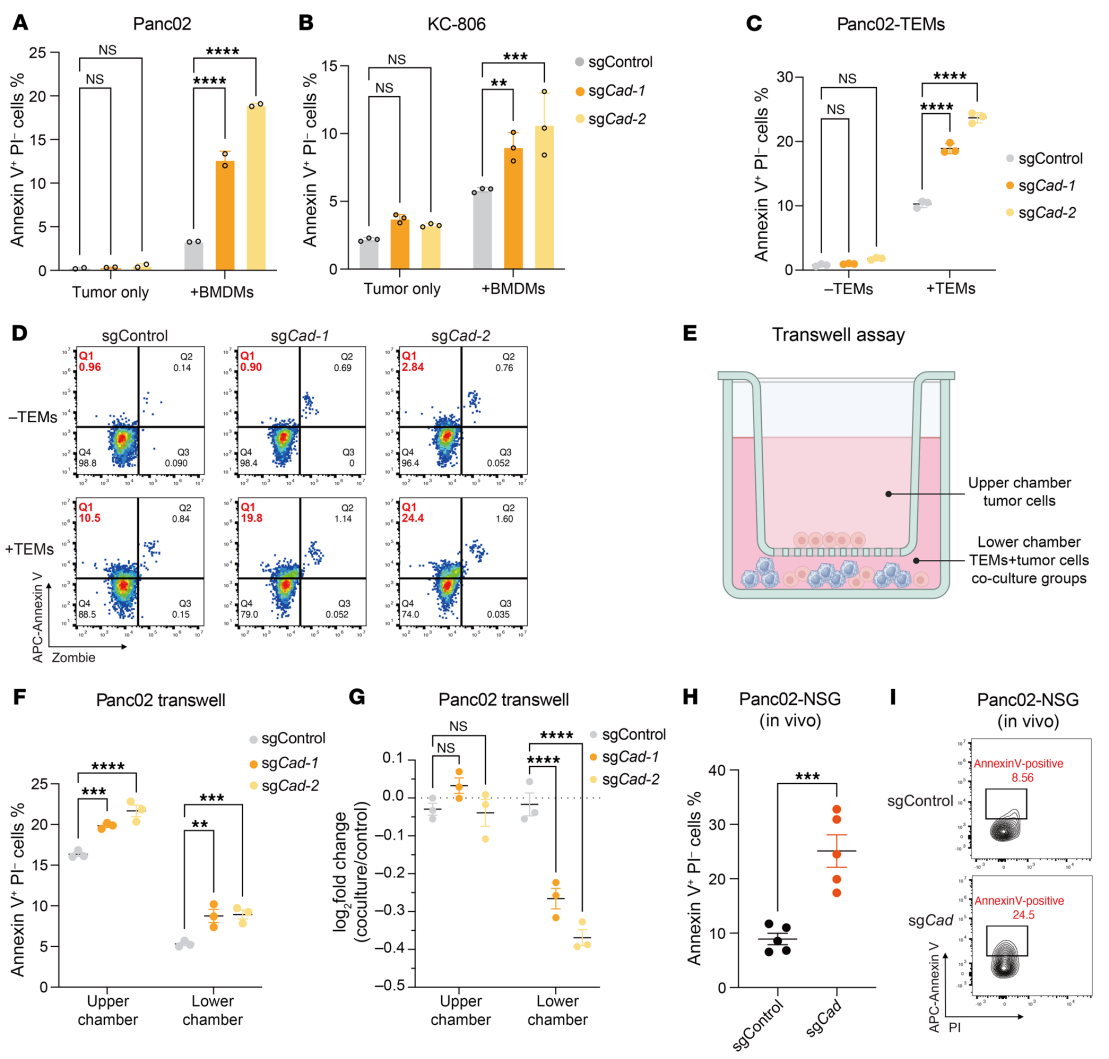
Inactivation of *Cad* increased the exposure of phosphatidylserine in the presence of macrophages. (**A** and **B**) Panc02 (**A**) or KC-806 (**B**) cells were either cultivated alone or cocultured with BMDMs for 24 hours. The levels of Annexin V^+^ PI^–^ cells were quantified by FACS. (**C** and **D**) Panc02 cells were either cultivated alone or cocultured with TEMs. The levels of Annexin V^+^ Zombie^–^ cells were quantified by FACS. Percentage of Annexin V^+^ Zombie^-^ cells (**C**) and representative FACS plots (**D**) are shown. (**E**) Schematic illustration of the macrophage-Panc02 Transwell coculture assay. Created using BioRender.com. (**F**) Control or *Cad-*KO Panc02 cells were cultured as described in **E** at an E/T ratio of 2:1 for 24 hours. The percentage of Annexin V^+^ Zombie^–^ tumor cells in both the upper and lower chambers was quantified by FACS. (**G**) Control or Cad-KO Panc02 cells (CFSE^+^) were mixed with WT Panc02 cells and then cultured as described in **E** at an E: T ratio of 4:1 for 24 hours. Log_2_ fold changes of the percentage of KO cells in both chambers are shown. (**H** and **I**) In vivo quantification of Annexin V^+^ PI^–^ tumor cells in control or *Cad*-KO Panc02 tumors. Statistical analysis (**H**) and representative FACS plots (**I**) are shown. For **A**–**C**, **F**, and **G**, data are represented as mean ± SD and analyzed by 2-way ANOVA. For **H**, data are represented as mean ± SEM and analyzed by unpaired *t* test. **P < 0.01, ***P < 0.001 and ****P < 0.0001. All data are representative of at least 2 independent experiments.

Next, we investigated the underlying mechanisms making these KO cells more susceptible to phagocytosis. To this end, we analyzed the expression levels of known eat-me and do-not-eat-me signals. Inactivation of de novo pyrimidine synthesis did not affect CD47 levels on the cell surface (Supplemental Figure 4A), indicating that the *Cad* pathway influences macrophage phagocytosis through a CD47-independent mechanism. Intriguingly, in *Cad*-KO cells, but not control Panc02 or KC-806 cells, coculture with BMDMs resulted in substantially increased Annexin V staining, indicating elevated externalization of PtdSer, a principal eat-me signal (Figure 4, A and B, and Supplemental Figure 4B). Similarly, when Panc02 cells were cocultured with TEMs, we observed substantially higher levels of Annexin V^+^ cells (Figure 4, C and D). Notably, while these cells were Annexin V^+^, they remained negative for cell death markers such as propidium iodide (PI) (Supplemental Figure 4B) or Zombie dye (Figure 4D) staining. We also assessed the proliferation rates of *Cad*-KO and control Panc02 and KC-806 cells and found no differences (Supplemental Figure 4, C–E). Additionally, *Cad* KO did not trigger caspase-3/7 activation (Supplemental Figure 4, F and G) or reduce mitochondrial membrane potential as determined by CMXRos (Supplemental Figure 4, H and I). These data together indicate that while the presence of macrophages induces the externalization of PtdSer in *Cad*-KO cells, these cells were alive and not yet apoptotic.

To further investigate whether macrophage-mediated phagocytosis is necessary to eliminate *Cad*-KO tumor cells in vitro, we designed a Transwell experiment in which BMDMs were cocultured with a mixture of *Cad*-KO and control tumor cells in the lower chamber, while only tumor cells were present in the upper chamber (Figure 4E). This setup ensured that only the tumor cells in the lower chamber could directly interact with macrophages. Enhanced Annexin V staining was observed in *Cad*-KO cells in both upper and lower chambers, again indicating that the presence of macrophages could trigger enhanced externalization of PtdSer in *Cad*-KO cells (Figure 4F). Interestingly, the proportion of Annexin V^+^ cells was much lower in the lower chamber, where tumor cells were cocultured with macrophages, suggesting that Annexin V^+^ cells were likely eliminated by macrophages through phagocytosis (Figure 4F). In terms of tumor cell number, only the *Cad*-KO cells were depleted in the lower chamber, indicating that direct contact with macrophages is required for their elimination (Figure 4G). These data indicate that macrophages are not only necessary to induce the PtdSer externalization but also required to eliminate *Cad*-KO cells. To further examine the relevance of this finding in vivo, we stained *Cad*-KO and control Panc02 tumor cells (Td-Tomato^+^) with Annexin V. Consistent with the in vitro findings, *Cad* KO tumor cells exhibited higher levels of Annexin V^+^ cells (gating based on PI-negative cells) compared with control tumor cells (Figure 4, H and I, and Supplemental Figure 4J). Together, these data suggest that *Cad* KO also resulted in enhanced levels of externalized PtdSer in vivo.

### Macrophages suppress the UMP salvage pathway and increase the dependency of de novo pyrimidine synthesis in tumor cells.

**Figure 5:**
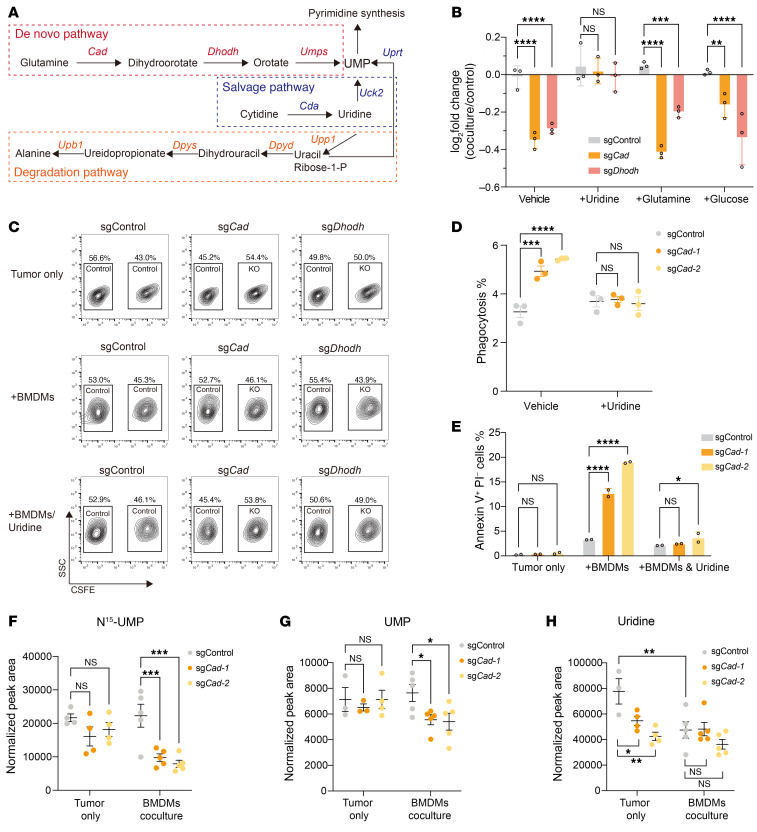
Macrophages suppress the UMP salvage pathway in tumor cells. (**A**) Depiction of de novo synthesis, salvage, and degradation pathways of UMP. (**B** and **C**) Tumor-macrophage coculture competition experiments, with the addition of indicated metabolites to the culture medium at 200 μM, including uridine, glutamine, and glucose. Control Panc02 cells were mixed with CSFE-labeled cells transduced with sgRNA targeting indicated genes. The cell mixtures were then cocultured with BMDMs. Log_2_ fold changes of the percentage of KO cells upon coculture with BMDMs are shown in **B**. Representative FACS results are presented in **C**. (**D**) Phagocytosis assay of BMDMs cocultured with CFSE-labeled control or *Cad*-KO Panc02 cells in the presence or absence of supplemented uridine (200 μM). The percentages of phagocytosis (F4/80^+^CSFE^+^) were quantified by FACS. (**E**) Control or *Cad*-KO Panc02 cells were either cultivated alone or cocultured with BMDMs in the presence or absence of uridine (200 μM). The percentage of Annexin V^+^ PI^–^ cells was quantified by FACS. (**F**–**H**) Control or *Cad*-KO Panc02 cells were either cultured alone (tumor only) or cocultured with BMDMs for 24 hours, and then tumor cells were sorted for mass spectrometry analysis. Normalized peak area of labeled N^15^-UMP (**F**), unlabeled UMP (**G**), or uridine (**H**) are presented. Data are represented as mean ± SD and analyzed by 2-way ANOVA (**B** and **D**–**H**). *P < 0.05, **P < 0.01, ***P < 0.001, ****P < 0.0001. All data are representative of at least 2 independent experiments.

We then explored the downstream mechanisms of how macrophages interact with the de novo pyrimidine synthesis pathway in tumor cells. UMP can be synthesized through two distinct routes: the de novo pathway, which relies on the enzymes *Cad*, *Dhodh*, and *Umps*, and the salvage pathway, which converts uridine or uracil into UMP (Figure 5A). The *Cad*-KO cells are defective for the de novo pathway whereas the UMP salvage pathway is not affected. Notably, supplementing uridine or cytidine in the coculture assays completely reversed the phagocytosis susceptibility observed in *Cad-* and *Dhodh*-KO cells (Figure 5, B–D, and Supplemental Figure 5, A–C), suggesting that the heightened sensitivity to phagocytosis is linked to a defect in UMP salvage pathway: conversion of uridine to UMP. Consistently, the level of Annexin V^+^ cells upon coculture with macrophages could also be rescued by the addition of uridine (Figure 5E).

To directly investigate whether macrophages could influence UMP synthesis in tumor cells, we measured UMP from de novo synthesis pathway by tracing the incorporation of amide-15N-labeled glutamine into UMP in Panc02 cells. We observed a reduction of N^15^-labeled UMP in *Cad*-KO cells upon coculture with BMDMs, confirming the critical role of *Cad* in de novo UMP synthesis in the presence of macrophages (Figure 5F). Next, we assessed the total UMP levels using mass spectrometry in Panc02 cells under regular culture conditions (tumor only) or coculture with BMDMs. First, in the absence of BMDMs (tumor-only condition), there was no significant change in UMP between control and *Cad-*KO Panc02 cells (Figure 5G), suggesting that the salvage pathway is sufficient to compensate for UMP level when de novo synthesis pathway is inactivated. In contrast, when *Cad*-KO cells were cocultured with BMDMs, the total UMP levels were dramatically reduced, indicating that macrophages could suppress the UMP salvage pathway in tumor cells (Figure 5G). We next examined the level of uridine, which converts to UMP in the salvage pathway. In line with UMP data, we observed that uridine levels were substantially reduced in control Panc02 cells upon coculture with BMDMs, implying a reduced pool of uridine of the salvage pathway in generating UMP when macrophages are present (Figure 5H). Interestingly, in *Cad*-KO cells, the uridine levels were also reduced without BMDM coculture, potentially due to increased uridine consumption to maintain an adequate UMP pool when the de novo synthesis pathway is defective (Figure 5H). Uridine concentrations in the cell-culture supernatants showed no difference between control and *Cad* KO groups (Supplemental Figure 5, D and E), thereby ruling out reduced uridine supply as a potential cause of the UMP defect. Together, these data suggest that the presence of BMDMs suppresses the salvage pathways and reduces the UMP pool in tumor cells.

### Macrophages suppress UMP salvage pathway through the induction of Upp1, which facilitates the degradation of uridine.

**Figure 6:**
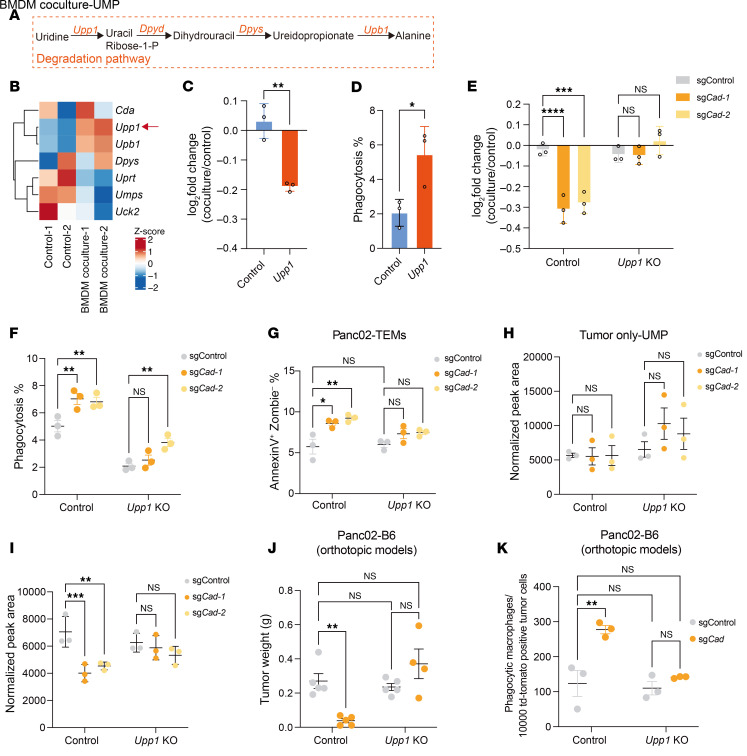
Macrophage-induced upregulation of *Upp1* in cancer cells enhances their reliance on the de novo pyrimidine synthesis pathway. (**A**) Depiction of degradation pathways of uridine. (**B**) Heatmap showing the expression level of genes related to UMP metabolism in *Cad*-KO Panc02 cells in the absence or presence of BMDMs. *Upp1* is highlighted. (**C**) Control Panc02 cells were mixed with CFSE-labeled control or *Upp1* overexpression Panc02 cells and cocultured with BMDMs for 24 hours. Log_2_ fold changes are shown. (**D**) CFSE-labeled Panc02 cells were cocultured with BMDMs for 24 hours, and the percentages of phagocytic BMDMs were quantified by FACS. (**E**) Panc02 tumor cells with indicated genes knocked out were labeled with pHrodo; then mixed with control cells under the same genetic background, respectively; and cocultured with BMDMs for 24 hours. Log_2_ fold changes are shown. (**F**) pHrodo-labeled Panc02 cells were cocultured with BMDMs for 24 hours, and percentages of phagocytic BMDMs were quantified by FACS. (**G**) Panc02 tumor cells with indicated genes knocked out were cocultured with TEMs 24 hours. The levels of Annexin V^+^ Zombie^–^ cells were quantified by FACS. (**H** and **I**) Metabolite analysis of UMP in *Cad* single-KO or *Cad/Upp1* double-KO Panc02 cells cultured without (**H**) or with (**I**) BMDMs for 24 hours. (**J**) 1 × 10^6^ Panc02-Td-Tomato cells with indicated genes knocked out were orthotopically implanted into B6 mice. Tumor weight was measured on day 34 after implantation. (**K**) Quantification of phagocytic macrophages as described in **J**. For **C**–**I**, data are represented as mean ± SD and analyzed by unpaired *t* test (**C** and **D**) or 2-way ANOVA (**E**–**I**). For **J** and **K**, data are represented as mean ± SEM and analyzed by 2-way ANOVA. *P < 0.05, **P < 0.01, ***P < 0.001, ****P < 0.0001. Data are representative of at least 2 independent experiments (**C**–**K**).

Next, we asked how macrophages suppress the UMP salvage pathway in tumor cells. To investigate this, we conducted RNA-Seq analysis on *Cad*-KO Panc02 tumor cells, which lack de novo synthesis, thus enabling us to examine compensatory effects from the UMP salvage pathway. These cells were either cultured alone or cocultured with BMDMs. GSEA analysis revealed that coculturing with macrophages induced substantial changes in gene expression in tumor cells, affecting many pathways, including upregulation of pathways associated with hypoxia and interferon response (Supplemental Figure 6, A and B). Interestingly, upon coculture with BMDMs, the expression of *Upp1*, the key enzyme that catalyzes the phosphorylation of uridine (or 2′-deoxyuridine) to uracil and ribose-1-phosphate (Figure 6A), was substantially upregulated upon coculture with BMDMs (Figure 6B and Supplemental Figure 6, A and B). We hypothesized that the upregulated UPP1 led to increased uridine catabolism and compromised the salvage pathway of UMP. Consistent with this hypothesis, overexpression of *Upp1* in Panc02 cells lead to enhanced sensitivity to macrophage-mediated phagocytosis (Figure 6, C and D, and Supplemental Figure 6C). Importantly, knocking out *Upp1* could fully rescue the sensitized phenotype to phagocytosis caused by the inactivation of *Cad*, suggesting that the increased level of UPP1 is responsible for enhanced sensitivity to phagocytosis in *Cad*-deficient cells (Figure 6, E and F, and Supplemental Figure 6, D and E). Consistently, the upregulation of Annexin V^+^/PI^–^ percentages in the *Cad*-deficient group were also restored by *Upp1* KO (Figure 6G). We then performed mass spectrometry to determine the level of UMP in WT and *Upp1*-deficient Panc02 cells. When tumors were cultured without BMDMs, we observed that *Upp1* KO did not substantially change UMP levels (Figure 6H). In contrast, in the presence of BMDMs, UMP levels of *Cad* KO groups were dramatically reduced (Figure 6I), aligning with our previous observations (Figure 5G), indicating that macrophages suppress the salvage pathway of UMP synthesis. Notably, *Upp1* KO completely reversed this effect, resulting in similar levels of UMP in both control and *Cad*-KO cells (Figure 6I).

To further validate whether *Upp1* KO could recapitulate the in vitro findings in vivo, we conducted in vivo orthotopic experiments using B6 mice. In line with the ex vivo data, *Upp1* KO mitigated the tumor growth suppression induced by *Cad* KO (Figure 6J and Supplemental Figure 6F). Additionally, the proportion of phagocytic macrophages was restored in *Upp1/Cad* double-KO (DKO) groups (Figure 6K and Supplemental Figure 6G). Collectively, these results suggest that the upregulation of *Upp1* is essential for promoting phagocytosis in *Cad*-KO tumor cells.

### Macrophage-released cytokines, including IL-1 and TNF-α, induce the expression of Upp1 in tumor cells.

**Figure 7:**
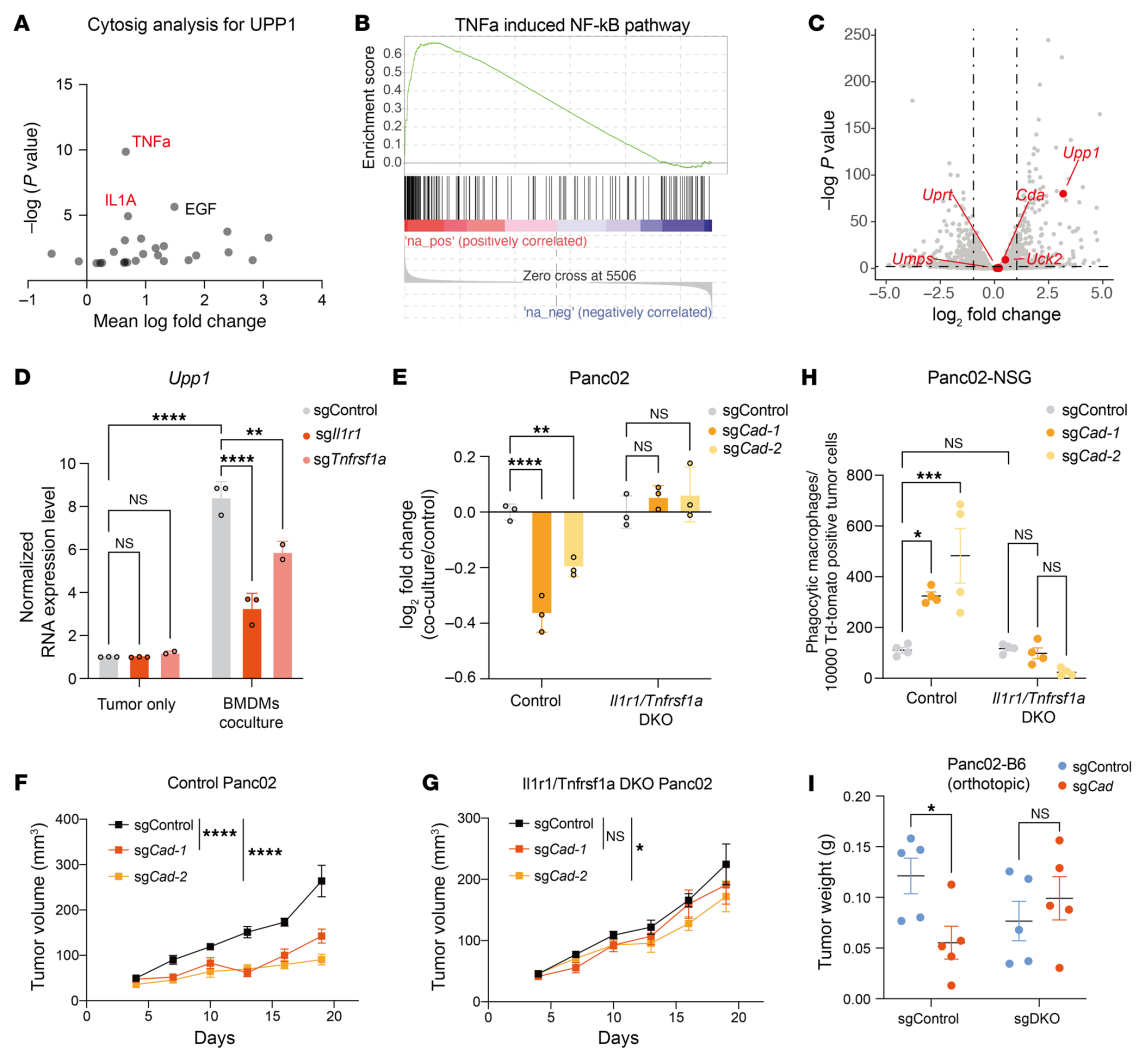
TNF-α and IL-1 are the key cytokines that induce the expression of *Upp1* to modulate the dependency on *Cad*. (**A**) CytoSig analysis of cytokines that are predicted to upregulate *Upp1* expression. (**B**) GSEA analysis of RNA-Seq data showing the enrichment of NF-κB pathway activation upon TNF-α treatment in Panc02 cells. (**C**) Volcano plot of RNA-Seq analysis showing the induction of *Upp1* in Panc02 cells upon treatment with TNF-α. Pyrimidine synthesis–associated genes are highlighted in red. (**D**) *Upp1* mRNA expression level, quantified by qPCR, in control, *Tnfrsf1a*-, or *Il1r1*-KO Panc02 cells in the presence or absence of BMDMs. (**E**) Control or *Tnfrsf1a*/*Il1r1* double-KO (DKO) Panc02 cells were transduced with control sgRNAs or sgRNAs targeting *Cad*. These cells (pHrodo^+^) were then mixed with control cells under the same genetic background and then cocultured with BMDMs for 24 hours for phagocytosis. Log_2_ fold change of the percentage of pHrodo^+^ cells upon coculture with BMDMs is presented. (**F** and **G**) Growth curves of control or *Cad*-KO conditions in parental Panc02 (**F**) or *Tnfrsf1a*/*Il1r1* double-KO Panc02 tumors (**G**) in NSG mice. (**H**) Quantification of phagocytic macrophages in Panc02-GFP tumors in experiments as described in **F** and **G**. TAMs were gated on F4/80 and Cd11b. Phagocytic macrophages were determined based on the double positive of F4/80 and GFP. (**I**) 1 × 10^6^ of Panc02 pancreatic cancer cells with indicated genes knocked out were orthotopically implanted into B6 mice. Tumor weight was measured on day 28 after implantation. For **D** and **E**, data are represented as mean ± SD and analyzed by 2-way ANOVA. For **F**–**I**, data are represented as mean ± SEM and analyzed by mixed-effects model (REML) test (**F** and **G**) or 2-way ANOVA (**H** and **I**). *P < 0.05, **P < 0.01, ***P < 0.001, ****P < 0.0001. Data are representative of at least 2 independent experiments (**D**–**I**).

To investigate the mechanisms by which macrophages stimulate *Upp1* expression in tumor cells, we utilized the CytoSig database (https://cytosig.ccr.cancer.gov/), which systematically assessed the cytokine signaling activity at tissue and single-cell levels (24). This resource enabled us to identify potential macrophage-released cytokines that might upregulate *Upp1* expression. CytoSig analysis revealed that genes encoding for proinflammatory cytokines, specifically *TNF* and *IL1A*, are likely to induce *Upp1* expression, as evidenced by RNA-Seq data from various cell lines (Figure 7A). By analyzing published single-cell RNA-Seq data, we confirmed that both TNF-α and IL-1 were highly expressed in TAMs and BMDMs, respectively (Supplemental Figure 7, A–D). To determine whether *Upp1* could be induced by TNF-α, we conducted RNA-Seq analysis on Panc02 cells with and without TNF-α treatment. TNF-α treatment induced a strong NF-κB gene expression signature as well as *Upp1* expression (Figure 7, B and C), consistent with previous literature indicating that the promoter of *Upp1* could be bound by NF-κB (25, 26). Coculture of BMDMs with tumor cells similarly upregulated *Upp1* expression in tumor cells (Figure 7D). Notably, this increase was substantially reduced when IL-1 or TNF-α receptors were genetically ablated (Figure 7D), underscoring the essential roles of TNF-α and IL-1 in *Upp1* upregulation in tumor cells cocultured with macrophages. To further confirm the importance of these cytokines in mediating tumor cell susceptibility to macrophage-induced phagocytosis, we knocked out either TNF-α receptor (*Tnfrsf1* KO) or IL-1 receptor (*Il1r1* KO) (Supplemental Figure 7E). Knocking out either of the receptors mitigated the increased sensitivity of *Cad*-KO cells to BMDM-induced phagocytosis (Supplemental Figure 7F). Remarkably, KO of both receptors (DKO) (Supplemental Figure 7E) completely abolished the sensitive phenotype (Figure 7E), suggesting that the presence of both cytokines is crucial for sensitizing Panc02 cells to macrophage-induced phagocytosis.

To explore the in vivo implications of the TNF-α/IL-1 link with the pyrimidine metabolism pathway, we inoculated NSG mice with *Cad*-KO and control cells in WT and DKO Panc02 cell backgrounds. Consistent with our previous findings, *Cad* KO substantially slower tumor growth in Panc02 tumor model (Figure 7F). In contrast, the DKO of TNF-α/IL-1 receptors rescued the phenotype in *Cad*-KO tumors, resulting in similar growth rates for both tumors (Figure 7G). Consistently, DKO of TNF-α/IL-1 receptors similarly rescued the enhanced phagocytosis levels in *Cad*-KO tumors in vivo (Figure 7H and Supplemental Figure 7G). As expected, single KO of each receptor could also partially rescue the phenotype in *Cad*-KO tumor (Supplemental Figure 7, H–J). Using orthotopic Panc02 model, DKO of TNF-α/IL-1 receptors consistently rescued the tumor growth suppression phenotype induced by *Cad* KO (Figure 7I). Taken together, our results demonstrate that signaling of TNF-α and IL-1 is crucial for the antitumor effect mediated by *Cad-*KO tumor cells.

### Pyrimidine metabolism in tumor cells interacts with macrophages in clinical datasets.

**Figure 8:**
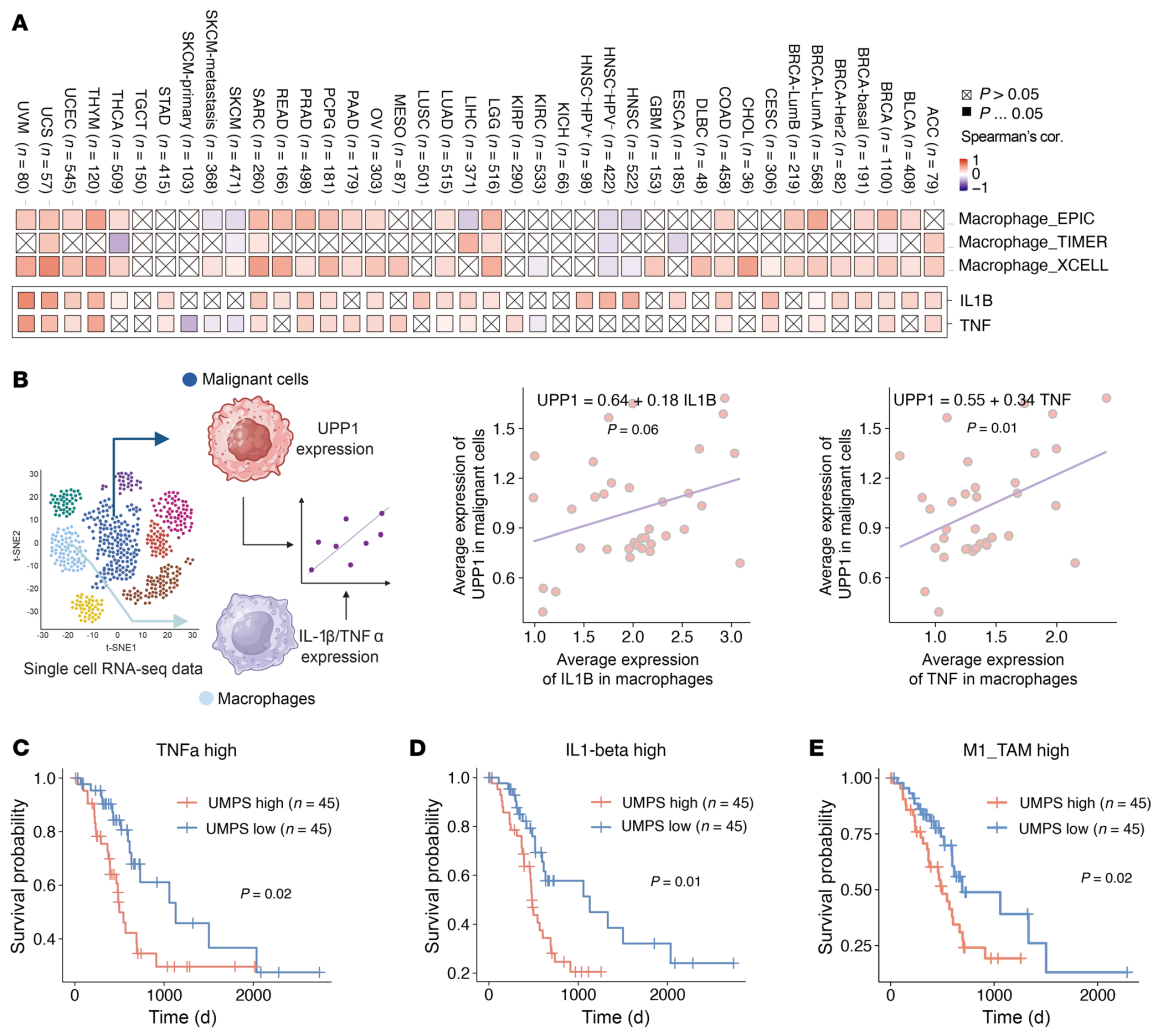
Human relevance for targeting de novo pyrimidine synthesis pathway in the presence of cytokine secrete macrophages. (**A**) Heatmap showing the correlation of UPP1 expression and macrophage infiltration (top panel) and the correlation of UPP1 expression and TNF and IL1B levels (bottom panel) in the TCGA cohort in TCGA bulk RNA-Seq datasets. (**B**) Single-cell analysis of the correlation between UPP1 expression level in tumor cells and TNF-α or IL-1β expression levels of macrophages in pancreatic cancer cohort. The left panel shows the strategy for single-cell analysis. Created using BioRender.com. The right panels show the correlation between UPP1 and TNF-α/IL-1β. (**C**–**E**) Overall survival of TCGA pancreatic adenocarcinoma based on the level of UMPS expression under the conditions of TNF-α expression level (**C**), IL-1β expression level (**D**), or estimated level of M1-like macrophage infiltration (**E**). Statistical analyses were performed by linear regression model (**B**) and log-rank test (**C**–**E**).

We evaluated the potential relationship among pyrimidine metabolism pathways, proinflammatory cytokines (TNF-α/IL-1), and macrophages across multiple pan-cancer The Cancer Genome Atlas (TCGA; https://www.cancer.gov/ccg/research/genome-sequencing/tcga) clinical data cohorts. We examined the association between the pyrimidine metabolism pathway and the estimated level of macrophages in bulk tumors. Overall, in most cancer types, the expression level of UPP1 is positively correlated with the estimated level of macrophage infiltration, as inferred by tumor deconvolution tools, including EPIC (27), TIMER (28), and CIBERSORT (29, 30) (Figure 8A). Additionally, the UPP1 level is also positively correlated with the levels of TNF-α and IL-1β in most cancer types (Figure 8A), consistent with our findings suggesting that both cytokines are required to upregulate UPP1 (Figure 7D). Since both tumor and immune cells express UPP1, we conducted further analyses using single-cell RNA-Seq datasets to delineate the source of UPP1 expression in each cell type within the tumor microenvironment. We performed a correlation analysis between the levels of TNF-α and IL-1β expression in macrophages and UPP1 levels in tumor cells (see Methods, Figure 8B). Consistent with our experimental data, the expression of UPP1 in tumor cells is positively correlated with the expression of both cytokines in macrophages (Figure 8B).

**Figure 9:**
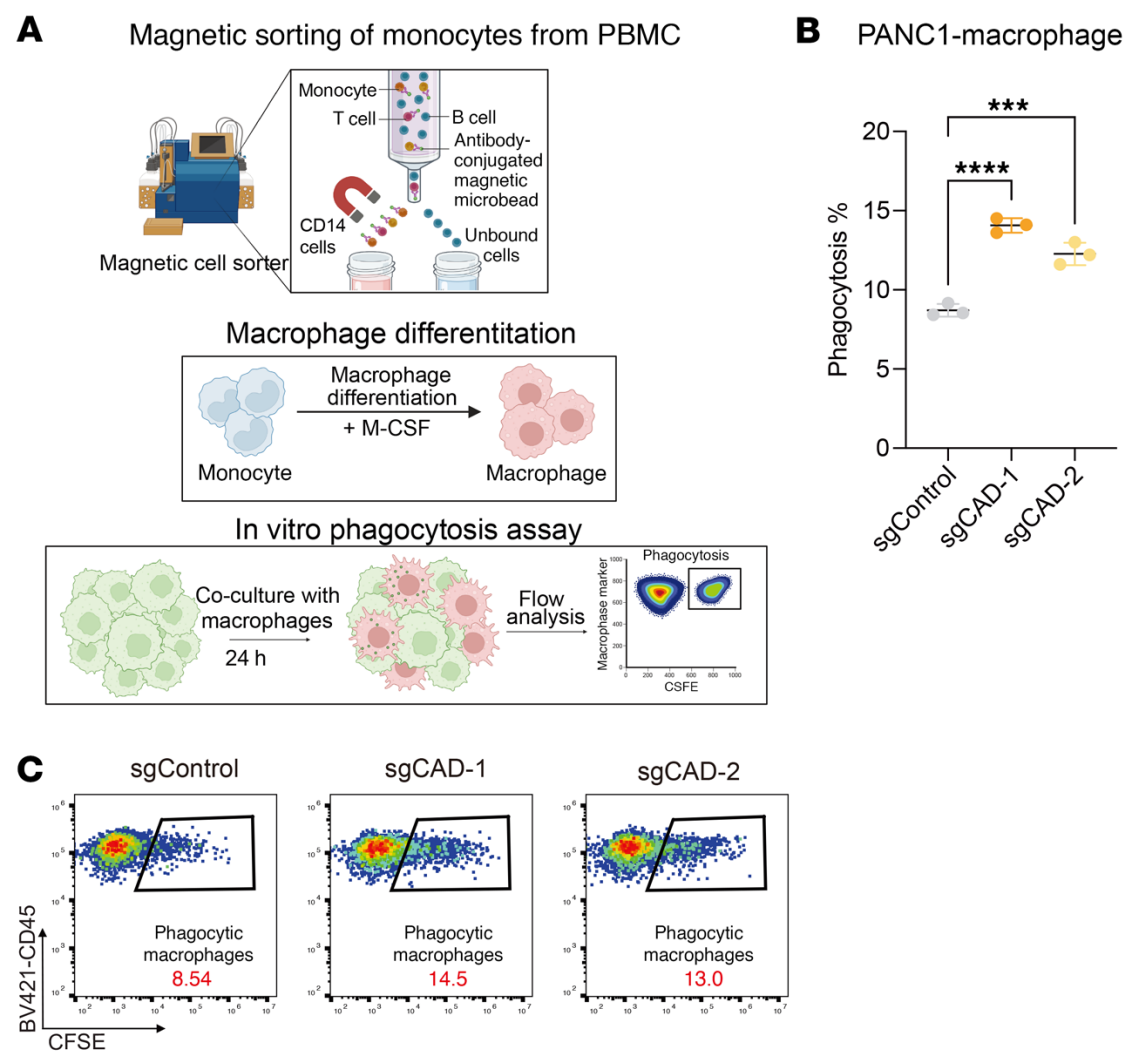
Loss of function of CAD sensitized pancreatic tumor cells to PBMC-derived macrophage-mediated phagocytosis. (**A**–**C**) In vitro phagocytosis assay by using human PBMC-derived macrophages. Monocytes were isolated from human PBMC and differentiated for 7 days. Coculture assays were then performed using monocyte-derived macrophages and CFSE-labeled human PANC1 cells. Phagocytosis percentages (CD45^+^CFSE^+^) were quantified by FACS. Statistical analysis of phagocytosis in the coculture assay is shown. (**A**) Illustration of phagocytosis assay performed by monocyte-derived macrophages. Created using BioRender.com. (**B**) Statistical analysis of phagocytosis in the coculture assay. (**C**) Representative flow plots of Figure 7B. For **B**, data are represented as mean ± SD and analyzed by 1-way ANOVA. ***P < 0.001, ****P < 0.0001. Data are representative of at least 2 independent experiments.

Next, we explored the interaction between the pyrimidine metabolism pathway and TNF-α/IL-1β expression levels. To this end, we divided patients into 4 groups based on their TNF-α/IL-1β and de novo pyrimidine synthesis gene expression levels. Overall, we found that patients with high TNF-α/IL-1β expression and low de novo pyrimidine synthesis gene expression, in particular UMPS, show better survival compared with the other groups (Supplemental Figure 8, A–F). We further analyzed the potential interaction between de novo pyrimidine pathway levels and cytokine expression levels. To this end, we examined a pancreatic cancer cohort for additional stratification into subgroups. We divided the patients into groups based on low or high expression of TNF-α/IL-1β or low or high levels of M1/M2 macrophage infiltration and examined the correlation of UMPS expression level with overall survival. Notably, low levels of UMPS expression were strongly correlated with survival benefits in patients with high TNF-α/IL-1β expression or in tumors heavily infiltrated with M1-like macrophages, where TNF-α and IL-1β are highly expressed (Figure 8, C–E, and Supplemental Figure 8, G–I), but not in tumors with high levels of M2-like macrophage infiltration (Supplemental Figure 8, J and K). However, these UMPS-associated survival benefits diminished in patients with low levels of TNF-α/IL-1β or M1-like macrophages (Figure 8, C–E, and Supplemental Figure 8, G–I). This finding aligns with our hypothesis that reduced pyrimidine synthesis may make tumors more susceptible to TNF-α/IL-1β^+^ macrophage-mediated immunity. Finally, we further validated our finding in a human system using primary human monocyte-derived macrophages cocultured with a PANC1 cell line. Consistent with murine data, *CAD* KO in PANC1 cells sensitized tumor cells to macrophage-mediated phagocytosis (Figure 9 and Supplemental Figure 8L). In summary, these data indicate that the de novo pyrimidine pathway in human pancreatic cancer cells modulates macrophage-mediated antitumor activity.

## Discussion

The molecular pathways that modulate macrophage-mediated phagocytosis of tumor cells remain an important topic that with important therapeutic implications. Prior studies have identified several tumor-intrinsic “phagocytosis checkpoints” that govern this process, including CD47, APMAP, CD24, SLAMF7, and PSGL1 (8, 10, 12, 31, 32). Additionally, activation of NF-κB signaling via cIAP1/2 antagonists has also been shown to induce macrophage phagocytosis and enhance antitumor immunity (33). In this study, we reveal a key crosstalk between tumor metabolism and macrophage response. Specifically, we demonstrate that disruption of key enzymes in the de novo pyrimidine synthesis pathway substantially boosts the phagocytosis of pancreatic cancer cells by macrophages. Mechanistically, we showed that such synthetic lethality is driven by macrophage-mediated suppression of the UMP salvage pathway through upregulation of the uridine phosphorylase *Upp1*, effectively reducing the uridine pool needed for UMP production — which become essential when the de novo pathway is inactivated in tumor cells. Importantly, our results demonstrate that either replenishing uridine, the substrate for *Upp1*, or knocking out *Upp1*, can reverse the heightened sensitivity of *Cad*-KO cells to macrophage-mediated phagocytosis, highlighting the essential role of *Upp1* in mediating the crosstalk between macrophages and tumor cells.

This mechanism diverges from traditional do-not-eat-me signals like CD47, suggesting an indirect role of the pyrimidine synthesis pathway in modulating the susceptibility to macrophage-mediated phagocytosis. Our results indicate that inhibiting this pathway induced the externalization of PtdSer, thereby facilitating the phagocytosis by macrophages. Importantly, in the absence of macrophages, inactivation of the same pathway (pyrimidine synthesis) does not induce externalization of PtdSer, suggesting a “synthetic lethality” model in which both macrophages and inactivation of pyrimidine synthesis pathway are required for elimination of tumor cells. However, since PtdSer is a major marker of apoptosis, it remains unclear whether PtdSer exposure alone is sufficient to induce macrophage-mediated phagocytosis or whether CAD inhibition triggers apoptosis that subsequently facilitates macrophage-mediated clearance of preapoptotic or apoptotic cells. This question remains to be clarified in future studies. IL-1β and TNF-α play crucial roles in modulating the tumor microenvironment and promoting tumor progression. For instance, TNF-α–secreting macrophages drive classical neoplastic cells into an aggressive phenotype through epigenetic regulation of the AP-1 transcriptional network (34). Additionally, neutrophil-derived TNF-α has been identified as a central regulator of T cell exhaustion through the CXCL-1 chemokine axis (35). IL1^+^ TAMs represent a major TAM subtype and are involved in the development of various cancer types (36). A recent study shows that IL-1β–producing TAMs can be elicited by prostaglandin E2 and TNF-α presented in the tumor microenvironment (3). These IL-1β^+^ TAMs, enriched in hypoxic regions of the tumor, further promote PDAC progression. Collectively, these findings highlight the protumoral roles of TNF-α– and IL-1β–expressing macrophages and their interdependent relationship. In our study, we demonstrate that TNF-α and IL-1β also reprogram the metabolic landscape of pancreatic cancer cells, rendering their reliance on the de novo pyrimidine synthesis pathway. We also observed enhanced phagocytosis in vivo when *Cad* was inactivated. However, because macrophages readily engulf apoptotic cells and cell debris, it remains unclear whether macrophages directly eliminate tumor cells through phagocytosis or whether *Cad* inactivation first induces apoptosis, with subsequent clearance via phagocytosis. Furthermore, as other immune cell types such as T cells can also produce TNF-α, the contribution and crosstalk of these immune cells in regulating tumor pyrimidine metabolism remain to be further investigated.

DHODH inhibitors have been shown to induce ferroptosis in cancer cells and to exert antitumor effects in pancreatic cancer models (37, 38). Our study expands this paradigm by demonstrating that pharmacological blockade of de novo pyrimidine synthesis also enhances macrophage-mediated phagocytosis of tumor cells. By coupling a direct cytotoxic mechanism with an immunomodulatory effect, DHODH inhibition may offer dual antitumor benefits. These findings support the clinical evaluation of DHODH inhibitors, either as monotherapy or in combination with macrophage-activating agents, such as cIAP1/2 antagonists (38). More broadly, our study highlights a critical facet of the tumor-TAM interaction: while proinflammatory cytokines IL-1β and TNF-α promote tumor progression, they also create a metabolic vulnerability in pancreatic cancer cells by enforcing reliance on the de novo pyrimidine synthesis pathway. Therapeutically targeting this pathway has the potential to disrupt the protumor feedback loop and convert it into an antitumor loop driven by immune processes, such as TAM-mediated phagocytosis.

## Methods

### Sex as a biological variable

Our study examined male and female animals, and similar findings are reported for both sexes.

### Cell lines

Panc02 (RRID: CVCL_D627) and KC-806 cells were cultured in DMEM (Gibco, catalog C11995500BT) supplemented with 10% fetal bovine serum (Biological Industries, catalog 04-001-1ACS), 100 mg/mL penicillin, and 100 U/mL streptomycin (Gibco, catalog 15140-122). L929 cells were cultured in DMEM supplemented with 10% fetal bovine serum, 1% Glutamax (Gibco, catalog 35050061), 1 mM sodium pyruvate (Gibco, catalog 11360070), 1% HEPES (Gibco, catalog 15630-080), 100 mg/mL penicillin, and 100 U/mL streptomycin. All cells were cultured at 37°C in 5% CO_2_.

### Primary cultures

BMDMs were isolated from mouse bone marrow and differentiated using L929 cell culture supernatant-containing medium for 7 days. BMDM cells were cultured in DMEM supplemented with 20% fetal bovine serum, 25% L929 cell culture supernatant, 100 mg/mL penicillin, and 100 U/mL streptomycin.

### Animal studies

Eight- to 12-week-old C57BL/6J (RRID: IMSR_JAX:000664) or NSG mice (RRID: IMSR_JAX:005557) were used for all animal experiments. C57BL/6J and NSG mice were bred at the Laboratory Animal Resources Center of Tsinghua University. All mice were maintained in pathogen-free facilities.

### Genome-wide tumor-BMDM coculture screen in Panc02 cells

#### Generation of Panc02 cells expressing inverted mIgG2a and Cas9.

Panc02 cells were transfected with PLVX-ZsGreen lentivirus expressing mIgG2a-Fc. These cells were then transfected with a lentivirus expressing Cas9 (RRID: Addgene_52962), followed by a 10-day selection phase in 5 μg/mL blasticidin (InvivoGen, catalog ant-bl-05).

#### Transduction with genome-wide sgRNA library (Brie).

We infected Panc02-mIgG2a-Cas9 cells with the Brie lentivirus library (Addgene #73633) at an approximate infection rate of 10%. Forty-eight hours after transfection, cells that had received the lentivirus were selected using 2 μg/mL puromycin (InvivoGen, catalog ant-pr-1) for a duration of 4 days.

#### Coculture experiments with BMDMs.

Ten days after viral transfection, tumor cells were divided into experimental and control groups, with 3 replicates in each. Each replicate consisted of 8 × 10^7^ cells, providing more than 1,000-fold coverage of the sgRNA library. In the experimental setup, tumor cells were cocultured with BMDMs at a 1:2 E/T ratio. Tumor cells in control group were incubated with conditioned media obtained from a coculture of BMDMs and tumor cells, aiming to minimize nonspecific effects. Following a 24-hour coculture period, the tumor cells were harvested using brief trypsinization. Genomic DNA was then extracted using the NucleoSpin Blood XL kit (MACHEREY-NAGEL, catalog 740950.50), according to the manufacturer’s instructions. PCR amplification of the sgRNA cassettes was conducted following the Broad GPP protocol (https://portals.broadinstitute.org/gpp/public/resources/protocols).

#### CRISPR screen data processing and analysis.

Fastq reads from the CRISPR screen were processed by MAGeCK (39) using the count module. The RRA module (default setting) was used to calculate log_2_ fold changes and *P* values of the genes. Custom R (v4.1.3) scripts were used to visualize the data.

### Generation of KO cell lines

sgRNA sequences used to generate KO cell lines are listed in Supplemental Table 1. For *Cad*, *Dhodh*, and *Umps*, sgRNAs were cloned into LentiCRISPRv2-hygro (RRID: Addgene_98291) and confirmed using Sanger sequencing. sgRNA constructs were cotransfected with psPAX2 (RRID: Addgene_12260) and pMD2.G (RRID: Addgene_12259) mixed with 1:4 PEI (YEASEN, catalog 40816ES02) in Opti-MEM (Gibco, catalog 31985070) for lentivirus production. The virus was harvested 48 hours after transfection and used to infect tumor cells. Hygromycin (200 μg/mL; InvivoGen, catalog ant-hg-1) was added to the culture medium for KO cell lines selection. KO efficiency of these cell lines was then verified via Western blot. To generate *Upp1*-KO, *Tnfrsf1a*-KO, and *Il1r1*-KO cell lines, sgRNAs were cloned into LentiCRISPRv2-EGFP, which was developed in-house by substituting the puromycin resistance gene with EGFP and verified using Sanger sequencing. Subsequently, the targeting and control sgRNAs were packaged into viruses to infect Panc02 tumor cells.

### Tumor-BMDM coculture competition assay

Target gene-KO cells were labeled with CFSE (Invitrogen, catalog C34554) or pHorodo Red (Invitrogen, catalog P36600) and then mixed with control cells at approximately 1:1 ratio on day 1. On day 2, a coculture consisting of 5 × 10^4^ BMDMs and 1.25 × 10^4^ tumor cell mixture was established in an untreated 6-well plate (NEST, catalog 703011). After 24 hours of coculture, all cells were harvested and stained with 0.1 mg/mL APC-F4/80 (BioLegend, catalog 123116) and 1 mg/mL DAPI (Cell Signaling Technology, catalog 4083S) in 50 μL FACS buffer for 20 minutes at room temperature. The cells were then resuspended in 100 μL FACS buffer and analyzed using FACS (CytoFLEX S, Beckman). The fold changes in the percentage of mutant tumor cells in the mixture, with and without the presence of BMDMs, were subsequently determined using the following formula: log_2_ fold changes (coculture/control) = log_2_ (percentage labeled tumor cells in coculture groups/percentage labeled tumor cells in the parallel tumor-only groups).

### Tumor-BMDM coculture phagocytosis assay

On day 1, target gene-KO cells were labeled with either CFSE (Invitrogen, catalog C34554) or pHorodo Red (Invitrogen, catalog P36600). The following day, 5 × 10^4^ BMDMs and 1 × 10^5^ tumor cells were cocultured in an untreated 6-well plate. After a 24-hour coculture period, all cells were harvested and stained with 0.1 mg/mL APC-F4/80 (BioLegend, catalog 123116) and 1 mg/mL DAPI (Cell Signaling Technology, catalog 4083S) in 50 μL of FACS buffer for 20 minutes at room temperature. Cells were then resuspended in 100 μL of FACS buffer and analyzed using FACS (CytoFLEX S, Beckman). The percentages of CFSE^+^ or pHorodo Red^+^ BMDMs in each group were subsequently determined.

### Generation of TEMs

For TEM generation, bone marrow cells were isolated and differentiated using MCSF-containing DMEM for 5 days. Subsequently, the culture medium was replaced with tumor conditional medium (the culture medium was prepared by harvesting the supernatant from tumor cells cultured for 2 days, followed by mixing it with an equal volume of MCSF containing DMEM supplemented with 20% fetal bovine serum) for 4 days. The cells were then cultured in this medium for an additional 4 days.

### Isolation of TAMs

To isolate TAMs, a pancreatic tumor model was generated by injecting AAV carrying sgRNAs targeting the tumor suppressor gene *p53* into LSL-Kras^G12D^; LSL-Cas9; Pdx1-Cre transgenic mice. Tumors were dissociated using a GentleMACS dissociator (Miltenyi Biotec) in a digestion solution containing 1 mg/mL collagenase type IV (Sigma-Aldrich, catalog C5138), 20 units/mL DNase type IV (Sigma-Aldrich, catalog C5205), and 0.1 mg/mL hyaluronidase type V (Sigma-Aldrich, catalog H6254) for 30 minutes at 37°C. The resulting cell suspension was passed through a 70 μm filter. Cells were first stained with the Zombie-NIR Fixable Viability Kit (BioLegend, catalog 423106) in PBS to assess viability, followed by anti-mouse CD16/32 staining (BioLegend, catalog 101320) to block the IgG Fc receptor. Subsequently, cells were stained with specific antibodies, including anti-mouse CD45 (BioLegend, catalog 103105), anti-mouse F4/80 (BioLegend, catalog 123116), and anti-mouse CD11b (BioLegend, catalog 101227). F4/80 and CD11b double-positive macrophages were then sorted using a BD AriaIII high-sensitivity flow cytometer. Isolated TAMs were cultured in DMEM supplemented with M-CSF and 20% fetal bovine serum.

### Tumor-TEM and -TAM coculture phagocytosis assay

To perform phagocytosis assay, on day 1, target gene-KO cells were labeled with CFSE (Invitrogen, catalog C34554). The following day, 5 × 10^4^ TEMs or TAMs and 1 × 10^5^ tumor cells were cocultured in an untreated 6-well plate. After a 24-hour coculture period, all cells were harvested and stained with 0.1 mg/mL APC-F4/80 (BioLegend, catalog 123116) and 1 mg/mL DAPI (Cell Signaling Technology, catalog 4083S) in 50 μL of FACS buffer for 20 minutes at room temperature. Cells were then resuspended in 100 μL of FACS buffer and analyzed using FACS (CytoFLEX S, Beckman). The percentages of CFSE^+^ TEMs or TAMs in each group were subsequently determined.

### Human macrophage phagocytosis coculture assay

To generate monocyte-derived macrophages, monocytes were isolated from 10 million human PMBCs (Milecell Biotechnologies, catalog PB010C) by using CD14 MicroBeads (Miltenyi Biotec, 130-050-201) as the instrument; then, CD14^+^ cells were separated by autoMACS Pro separators and cultured 7 days for macrophage maturation with 50 ng/mL of recombinant MCSF. To perform phagocytosis assay, on day 1, target gene-KO cells were labeled with CFSE (Invitrogen, catalog C34554). The following day, 1 × 10^4^ human macrophages and 2 × 10^4^ tumor cells were cocultured in an untreated 12-well plate. After a 24-hour coculture period, all cells were harvested and stained with 0.1 mg/mL BV421-CD45 (BioLegend, catalog 368522) and 1 mg/mL DAPI (Cell Signaling Technology, catalog 4083S) in 50 μL of FACS buffer for 20 minutes at room temperature. Cells were then resuspended in 100 μL of FACS buffer and analyzed using FACS (CytoFLEX S, Beckman). The percentages of CFSE^+^ TEMs or TAMs in each group were subsequently determined.

### Cell apoptosis assay

#### Annexin V staining.

Tumor cells (2.5 × 10^4^) and BMDMs (5 × 10^4^) or TEMs (5 × 10^4^) were cocultured in an untreated 6-well plate for 24 hours. To assess the Annexin V^+^ tumor cells after coculture with BMDMs or TEMs, the cells were first stained with 0.1 mg/mL BV421-CD45 (BioLegend, catalog 103133). For annexin V staining, the cells were processed using the APC Annexin V Apoptosis Detection Kit with PI (BioLegend, catalog 640932) or Zombie (Invitrogen, catalog L10119) based on the manufacturer’s instructions. Staining with APC-Annexin and PI/Zombie was performed for 15 minutes at room temperature in the dark. These samples were then analyzed on FACS.

#### Activated caspase-3/7 staining.

Tumor cells (2.5 × 10^4^) and BMDMs (5 × 10^4^) or TEMs (5 × 10^4^) were cocultured in an untreated 6-well plate for 24 hours. The cells were first stained with 0.1 mg/mL BV421-CD45 (BioLegend, catalog 103133), followed by staining with Annexin V and Zombie for 15 minutes, and finally with CellEvent Caspase-3/7 Green (Invitrogen, catalog C10427) based on manufacturer’s instructions. The prepared cells were then analyzed on FACS.

#### Mitochondria activity.

Tumor cells (2.5 × 10^4^) and BMDMs (5 × 10^4^) or TEMs (5 × 10^4^) were cocultured in an untreated 6-well plate for 24 hours. Cells were harvested and stained with 0.1 mg/mL APC-F4/80 (BioLegend, catalog 123116) in 50 μL FACS buffer for 20 minutes at room temperature. Then cells, were stained with MitoTracker Red CMXRos (Invitrogen, catalog M7512) at 37°C for 30 minutes. The prepared samples were then analyzed on FACS.

### Cell proliferation assay

For in vitro cell growth measurement, control or *Cad*-KO tumor cells were seeded at 5,000 cells per well in 12-well plate at day 0, and cell numbers were counted by FACS every 2 days. For growth competition assay, control or specific gene-KO tumor cells were labeled with CFSE and mixed with sgControl cells at 1:1, and the percentages of CFSE^+^ cells were measured by FACS daily.

### Real-time qPCR experiments

Total RNA was extracted using TRIzol reagent (Invitrogen, catalog 15596018), following the manufacturer’s protocol. From the extracted RNA, 1 mg was reverse-transcribed into cDNA using the HiScript III 1st Strand cDNA Synthesis Kit (Vazyme, catalog R312-01), in accordance with the manufacturer’s guidelines. The resulting cDNA samples were then diluted for use in real-time qPCR (RT-qPCR). For PCR amplification and detection, Taq Pro Universal SYBR qPCR Master Mix (Vazyme, catalog Q712-02) and gene-specific primers were employed, with the LightCycler480 (Roche) as the detection system. The RT-qPCR data were normalized against GAPDH mRNA levels. The primers used were as follows: Upp1-F: ACAGGAACTGAAGCAAAGGAC, Upp1-R: GTTGAAATGGTAGAGCACGTCTT; Gapdh-F: AGGTCGGTGTGAACGGATTTG, Gapdh-R: TGTAGACCATGTAGTTGAGGTCA.

### Western blot

Western blot was performed as previously described (40). Briefly, whole-cell lysates were solubilized in cell lysis buffer (Cell Signaling Technology, catalog 9803) supplemented with protease inhibitors (Sigma-Aldrich) and incubated at 4°C for 30 minutes. Following this, the lysates were centrifuged at 15,000*g* for 20 minutes. The supernatant was then collected for protein concentration determination. Protein concentrations were measured using the BCA Protein Assay (Thermo Fisher, catalog 23225). Subsequently, 20 μg of total protein from each sample was loaded onto 4%–12% gradient SurePAGE, Bis-Tris gels (GenScript, catalog M00654). After electrophoresis, gels were transferred onto Immobilon PVDF membranes (Millipore). Membranes were blocked in TBST containing 5% nonfat milk for 1 hour at room temperature, followed by overnight incubation with the specified antibodies, and then incubated with anti-rabbit HRP (Cell Signaling Technology, catalog 7074; RRID: AB_2099233). For detection, the blots were treated with Immobilon Western HRP substrate (Millipore), and chemiluminescence was captured using an Amersham Imager 600 (GE). The following antibodies were used: CAD (CST, catalog 93925S), GAPDH (CST, catalog 2118L), β-tubulin (CST, catalog 2146S), DHODH (Abcam, catalog ab174288), UMPS (Abclonal, catalog A13251), and UPP1 (Proteintech catalog 14186-1-AP).

### Mouse tumor experiments

To establish orthotopic models, 6- to 9-week-old mice were anesthetized with avertin and subjected to surgical procedures. After left abdominal incision, pancreatic tails were exposed and injected with 7 × 10^5^ tumor cells resuspended in cold PBS in a final volume of 30 μL. For macrophage depletion by aCSF1R antibody, the antibody was administered intraperitoneally at 200 μg. This was done 2 days before the tumor challenges and repeated every 3 days until the mice were sacrificed. The efficiency of macrophage depletion was assessed by FACS.

For macrophage depletion, either clodronate-liposome (Liposoma, catalog C-005) or control-liposome (Liposoma, catalog P-005) were administered intraperitoneally at 200 μL. This was done 2 days before the tumor challenges and repeated every 5 days until the mice were sacrificed. The efficiency of macrophage depletion was assessed by FACS. In the tumor challenge phase, either 2 × 10^6^ KC-806 or 2 × 10^6^ Panc02 cells, suspended in HBSS, were injected subcutaneously into the flanks of mice. The tumors’ length and width were measured every 3 days for NSG mice or every 4 days for WT B6 mice once they became palpable. Tumor volume was calculated using the formula (length × width^2^)/2. The endpoint was noted when the tumor diameter reached 2.0 cm or in the event of mouse mortality.

For BAY2402234 (MCE, catalog HY-112645) treatment experiments, 4 mg/kg BAY2402234 or an equivalent volume of vehicle (10% DMSO and 90% 20% SBE-β-CD in saline) was administered intratumorally every 3 days. Tumor dimensions were measured every 4 days for WT B6 mice, using the same palpability and calculation criteria as previously mentioned. The experiment was concluded under the same endpoint conditions. When measuring tumor sizes, investigators were blinded to sample allocations when feasible.

### Analysis of tumor-infiltrating immune cells

Tumors were dissociated using a GentleMACS dissociator with 1 mg/mL collagenase type IV (Sigma-Aldrich, catalog C5138), 20 units/mL DNAse type IV (Sigma-Aldrich, catalog C5205), and 0.1 mg/mL hyaluronidase type V (Sigma-Aldrich, catalog H6254) for 30 minutes at 37°C. The resulting cells were passed through a 70 μm filter, and a small fraction was reserved for FACS analysis. The cells were initially stained with the Zombie-NIR Fixable Viability Kit (BioLegend, catalog 423106) in PBS, followed by anti-mouse CD16/32 staining (BioLegend, catalog 101320) to block the Fc receptor of IgG. Subsequently, cells were stained with specific antibodies, including anti-mouse CD45 (BioLegend, catalog 103133), anti-mouse CD11b (BioLegend, catalog 101205) or anti-mouse CD11b (BioLegend, catalog 101207), anti-mouse F4/80 (BioLegend, catalog 123116), and anti-mouse Ly6C/G (BioLegend, catalog 108437). Data collection was performed using Beckman Coulter CytoFLEX S, and analysis was conducted with FlowJo (RRID: SCR_008520). To determine phagocytosis in vivo, normalized phagocytosis are calculated based on the following formula: phagocytic macrophages/10,000 Td-Tomato tumor cells = Td-Tomato + TAMs/Td-Tomato + tumor cells × 10,000. This formula normalizes the phagocytosis rate by accounting for the purity of Td-Tomato^+^ tumor cells in the tumors. Extremely small tumors (e.g., tumor weight <0.05 g) were excluded from TIL analysis due to low numbers of cells recovered.

### Bulk RNA-Seq

Panc02 cells were washed with PBS and lysed using TRIzol (Invitrogen, catalog 15596026) for RNA extraction. Subsequent RNA sequencing involved aligning the reads to the mouse reference genome mm10 using STAR (RRID: SCR_004463). FeatureCount was utilized to map aligned reads to genes and generate a gene count matrix. Differential gene expression analysis was performed using the DESeq2 R package (RRID: SCR_000154).

### Steady-state metabolite analysis

3 × 10^5^ BMDMs were plated overnight, followed by the addition of 1.5 × 10^5^ *Cad*-KO tumor cells for a 24-hour coculture period. After coculture, cells were harvested by trypsinization, stained with APC-F4/80 (BioLegend, catalog 123116), and F4/80^–^ tumor cells were sorted via FACS. The sorted tumor cells were then resuspended in 1 mL cold 80% methanol (Fisher chemical, catalog A452-4) and incubated overnight at –80°C. The next day, after centrifugation to remove debris, 1 mL of the supernatant was transferred to a new tube, dried in a SpeedVac (Labconco Centrivap), and stored at –80°C until LC-MS analysis.

For the analysis of metabolites in cell culture supernatants, 400 μL of the supernatant was mixed with 1.6 mL of methanol (Fisher Chemical, catalog A452-4) and incubated overnight at –80°C. Following a similar procedure of centrifugation and supernatant transfer, samples were dried in a SpeedVac and stored at –80°C until LC-MS analysis. These experiments were conducted in triplicate. Data normalization employed MinMax normalization across all measured metabolites in each sample. The abundance of each metabolite was then calculated relative to the control sample.

### 15N-amide glutamine isotope labeling through pyrimidine metabolism pathway

To assess the contribution of macrophages to the de novo pathway in UMP biosynthesis, 15N-glutamine tracing experiments were conducted with Panc02 control and *Cad*-KO cells. 3 × 10^5^ BMDMs were plated overnight prior to the addition of *Cad*-KO tumor cells for a 24-hour period. The following day, the cells were washed twice with glutamine-free media, and 1.5 × 10^5^ tumor cells were added. These cocultured cells were then maintained in glutamine-free media for 24 hours, subsequently supplemented with either 2 mM 15N-glutamine (MCE, catalog HY0390S9) or 2 mM Glutamine (Gibco Inc, catalog 25030081) for another 24 hours. Finally, tumor cells were isolated via FACS and converted into dried pellets, following the procedure outlined in *Steady-state metabolite analysis*.

### Single-cell analysis for human samples

Human scRNA-Seq datasets were sourced from the public database TISCH (http://tisch.compbio.cn/home/) (41). Within each dataset, malignant cells with 0 count for UPP1 expression and macrophages with 0 count for IL1B and TNF were excluded. Subsequently, the average expression levels of UPP1 in malignant cells and TNF/IL1B in macrophages were computed. The correlation between UPP1 expression in malignant cells and TNF/IL1B expression in macrophages was analyzed using linear regression in R, employing the lm() function. The specific dataset used and corresponding cell numbers are summarized in Supplemental Table 2.

### Analysis of TCGA cohorts

Transcriptome and clinical data were sourced from the TCGA Data Portal (https://www.cancer.gov/tcga). We selected all 33 cancer types for which transcriptome data were available for cancer samples. For this study, only samples categorized clinically as “primary tumor” were utilized. The median expression level served as the cutoff to categorize samples into either high expression or low expression groups. Survival analysis was performed using the survival package.

Genes used for generating M1-like macrophage signature were as follows: *IL23*, *TNF*, *CXCL9*, *CXCL10*, *CD86*, *IL1A*, *IL1B*, *IL6*, *CCL5*, *IRF5*, *IRF1*, *CD40*, *IDO1*, *KYNU*, and *CCR7*. Genes used for generating M2-like macrophage signature were as follows: *IL4R*, *CCL4*, *CCL13*, *CCL20*, *CCL17*, *CCL22*, *CCL24*, *LYVE1*, *VEGFA*, *VEGFB*, *VEGFC*, *VEGFD*, *EGF*, *CTSA*, *CTSB*, *CTSC*, *CTSD*, *TGFB1*, *TGFB2*, *TGFB3*, *MMP14*, *MMP19*, *MMP9*, *CLEC7A*, *WNT7B*, *FASL*, *TNFSF12*, *TNFSF8*, *CD276*, *VTCN1*, *MSR1*, *FN1*, and *IRF4*.

### Statistics

Statistical analyses were conducted using GraphPad Prism 10 software. Depending on the context, 2-tailed unpaired Student’s *t* tests, 1-way ANOVA, 2-way ANOVA or mixed-effects model (restricted maximum likelihood [REML]) tests were applied. For power analysis, the group sizes for in vivo experiments were determined empirically, drawing on prior experience with the respective tumor models. Similarly, group sizes for in vitro experiments were established based on our previous understanding of the variability in these experiments. *P* values of less than 0.05 were considered significant.

### Study approval

Approval for use of animals in experimentation was granted by the Association for Assessment and Accreditation of Laboratory Animal Care International and the Institutional Animal Care and Use Committee of Tsinghua University under protocol 19-PD-1.

### Data availability

All data generated or analyzed during this study are included in this article, its supplemental information files, and the Supporting Data Values file. RNA-Seq data in this publication have been deposited in the NCBI Gene Expression Omnibus (GEO; GSE262721).

## Author contributions

JZ provided conceptualization, conducted experiments, analyzed data, and wrote the original draft of the manuscript. PR analyzed data and wrote the original draft of the manuscript. Xinghao Li, Xinyu Li, YW, HG, LW, JH, SW, and ZG conducted experiments and analyzed data. MC provided conceptualization and provided reagents. ZZ provided conceptualization and reviewed and edited the manuscript. DP provided conceptualization, analyzed data, reviewed and edited the manuscript, and supervised of the project.

## Funding support

- National Natural Science Foundation of China grants, 82341026 and 82073163, to DP.
- National Key Research and Development Program of China, 2022YFC2505400, to DP.
- Tsinghua University Initiative Scientific Research Program, to DP.
- Tsinghua-Peking Joint Centre for Life Sciences, to DP and ZZ.

## Supplementary Material

Supplemental data

Unedited blot and gel images

Supporting data values

## Version 1 11/17/2025

Electronic publication
