# Supplementary material for: Targeting plasticity in the pyrimidine synthesis pathway potentiates macrophage-mediated phagocytosis in pancreatic cancer models
Source: J Clin Invest. 2025 Nov 17;135(22):e193370. doi: 10.1172/JCI193370 (PMC12618067; doi:10.1172/JCI193370)
Supplement: Supplemental data [file jci-135-193370-s215.pdf]

Supplementary Materials for

Targeting plasticity in the pyrimidine synthesis pathway potentiates macrophage-mediated phagocytosis in pancreatic cancer models

Authors: Jie Zhao[1,2], Xinghao Li[1], Xinyu Li[1], Pengfei Ren[3], Yilan Wu[1], Hao Gong[1], Lijian Wu[1], Junran Huang[1], Saisai Wang[1], Ziwei Guo[1], Mo Chen[1]*, Zexian Zeng[2,3]* and Deng Pan[1,2]*

Affiliations:

[1] Department of Basic Medical Sciences, State Key Laboratory of Molecular Oncology, Tsinghua University, Beijing, China

[2] Tsinghua-Peking Joint Centre for Life Sciences (CLS)

[3] Center for Quantitative Biology, Academy for Advanced Interdisciplinary Studies, Peking University, Beijing, China

Supplementary Figures

Figure S1

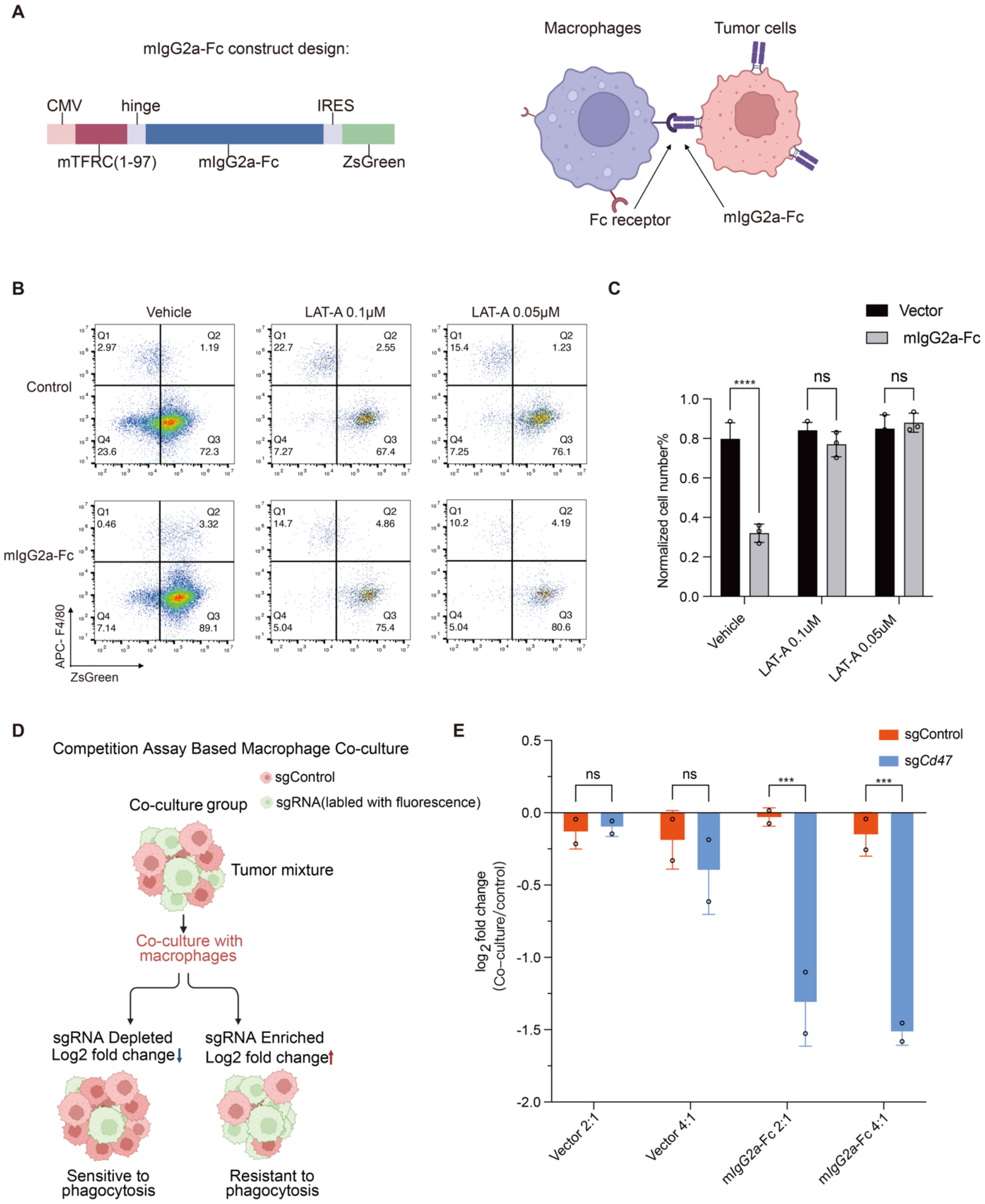

Figure S1. Establishing a robust *ex vivo* model for detection of Fc-mediated phagocytosis regulators in tumor cells, Related to Figure 1.

(**A**) Schematic presentation showing the design of construct expressing a mIgG2a-Fc domain following a type II transmembrane domain from mouse transferrin receptor (mTFRC) (left panel). The mIgG2a-Fc triggers Fc receptor-mediated phagocytosis by macrophages (right panel).

(**B-C**) BMDMs and Panc02-Fc cells were co-cultured, followed by the addition of Latrunculin A (LAT-A) at indicated concentrations to suppress BMDMs phagocytosis for a period of 24 hours. Subsequently, the number of surviving Panc02-Fc cells was quantified using FACS. The left panel shows the representative FACS results (**B**). The right panel summarized the normalized percentage of tumor cells left after co-culture with BMDMs (**C**).

(**D**) Illustration of the *in vitro* competition assay. Tumor cells with targeted gene knockout (e.g. CD47; Td-Tomato^{+}), were mixed with control tumor cells at approximately 1:1 ratio. After co-culture with BMDMs for 24 hours, the proportion of Td-Tomato^{+} cells were quantified using FACS. Created using BioRender.com.

(**E**) *In vitro* competition assay of co-culture experiments consisting of BMDMs and Panc02 tumor cells with or without the expression of Fc fragment. CD47 knockout Panc02 cells (sg*Cd47*) were mixed with control sgRNA cells (sgControl) and then co-cultured with BMDMs for 24 hours with indicated effector-to-target (E: T) ratios. The $\log_2$ fold changes of the percentage of KO cells (Td-Tomato^{+}) relative to the total Panc02 cells, comparing in the absence and presence of BMDMs, are shown.

Data are represented as mean ± SD (**C, E**), and analyzed by two-way ANOVA. $^{***}P < 0.001$ and $^{****}P < 0.0001$. Data are representative of at least 2 independent experiments (**C, E**).

Figure S2

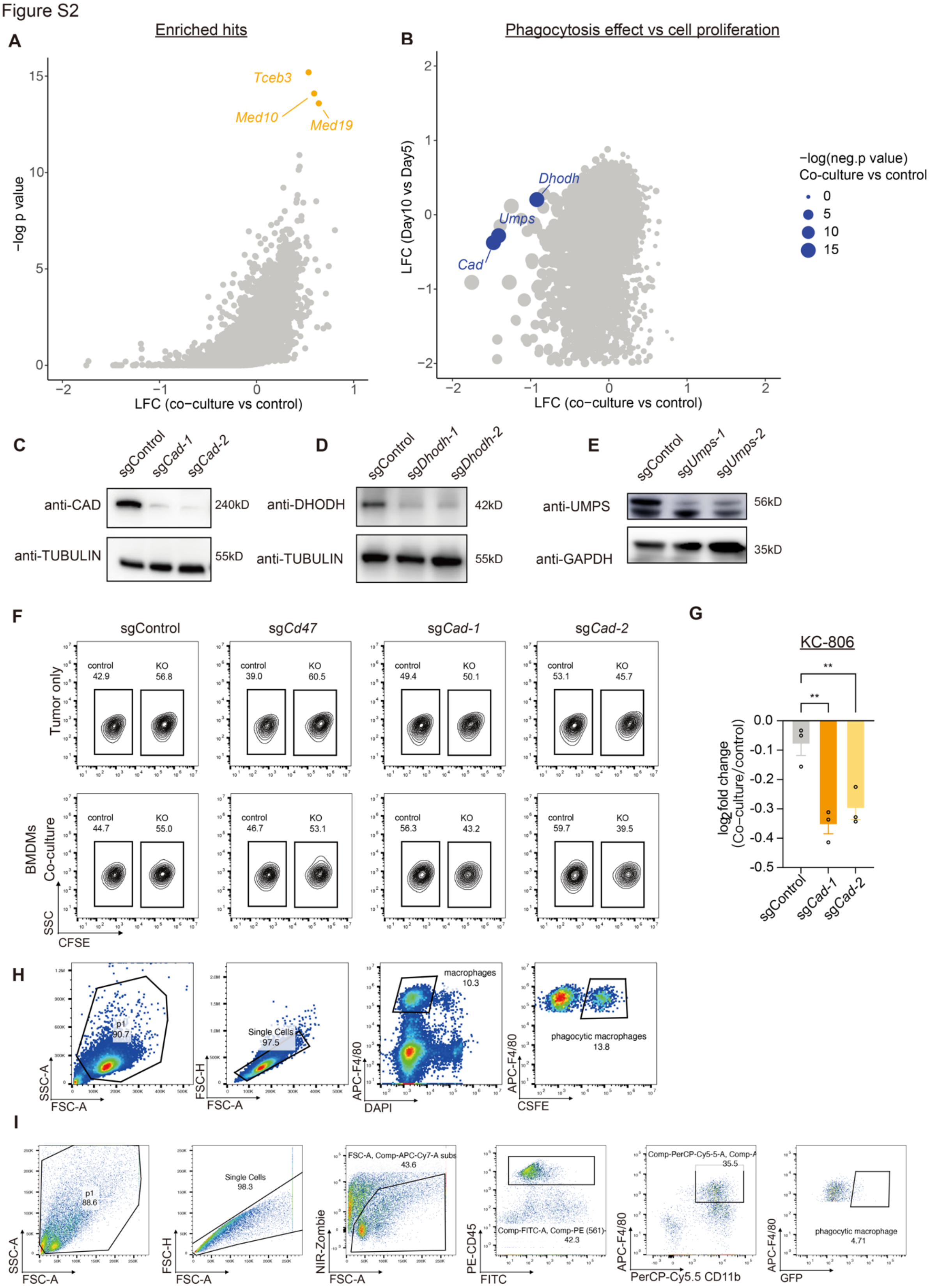

Figure S2. Inactivation of *de novo* pyrimidine synthesis pathway sensitized pancreatic

cancer cells to macrophage-mediated phagocytosis, Related to Figure 1.

(**A**) Scatterplot showing the top enriched sgRNAs based on mean $\log_2$ fold change of sgRNA counts in BMDMs co-culture condition versus control condition.

(**B**) Scatterplot showing the $\log_2$ fold change of sgRNAs' effect on BMDM-mediated phagocytosis versus cell proliferation (Day 10 vs Day 5 upon transduction of sgRNA library). Genes related to *de novo* pyrimidine synthesis pathway were highlighted.

(**C-E**) Western blot analysis of knockout efficiency of *Cad* (**C**), *Dhodh*(**D**) and *Umps* (**E**) in Panc02-Fc cells.

(**F**) *In vitro* competition assay of Panc02 cells co-cultured with BMDMs. Representative FACS plot of Figure **1E**, showing the depletion of cells transduced with indicated sgRNAs upon co-culture with BMDMs.

(**G**) *In vitro* competition assay based on co-culture of BMDMs and KC-806 tumor cells. Control KC-806 cells were mixed with CFSE-labelled cells transduced with control sgRNA, or sgRNA targeting indicated genes. The cell mixtures were then co-cultured with BMDMs for 24 hours. Log_2 fold change of the percentage of KO cells upon co-culture with BMDMs was shown.

(**H**) Gating strategy of *in vitro* phagocytosis assay. Phagocytic macrophages were determined based on double positive for F4/80 and labeled fluorescence signal.

(**I**) Gating strategy of tumor-associated macrophages (TAMs) sorted for *in vitro* phagocytosis assay in Figure **1I**. Tumor-associated macrophages were determined based on double positive for F4/80 and CD11b.

Data are represented as mean ± SD and analyzed by one-way ANOVA(**G**). $^{**}P < 0.01$. Data are representative of at least 2 independent experiments (**C-E, G**).

Figure S3

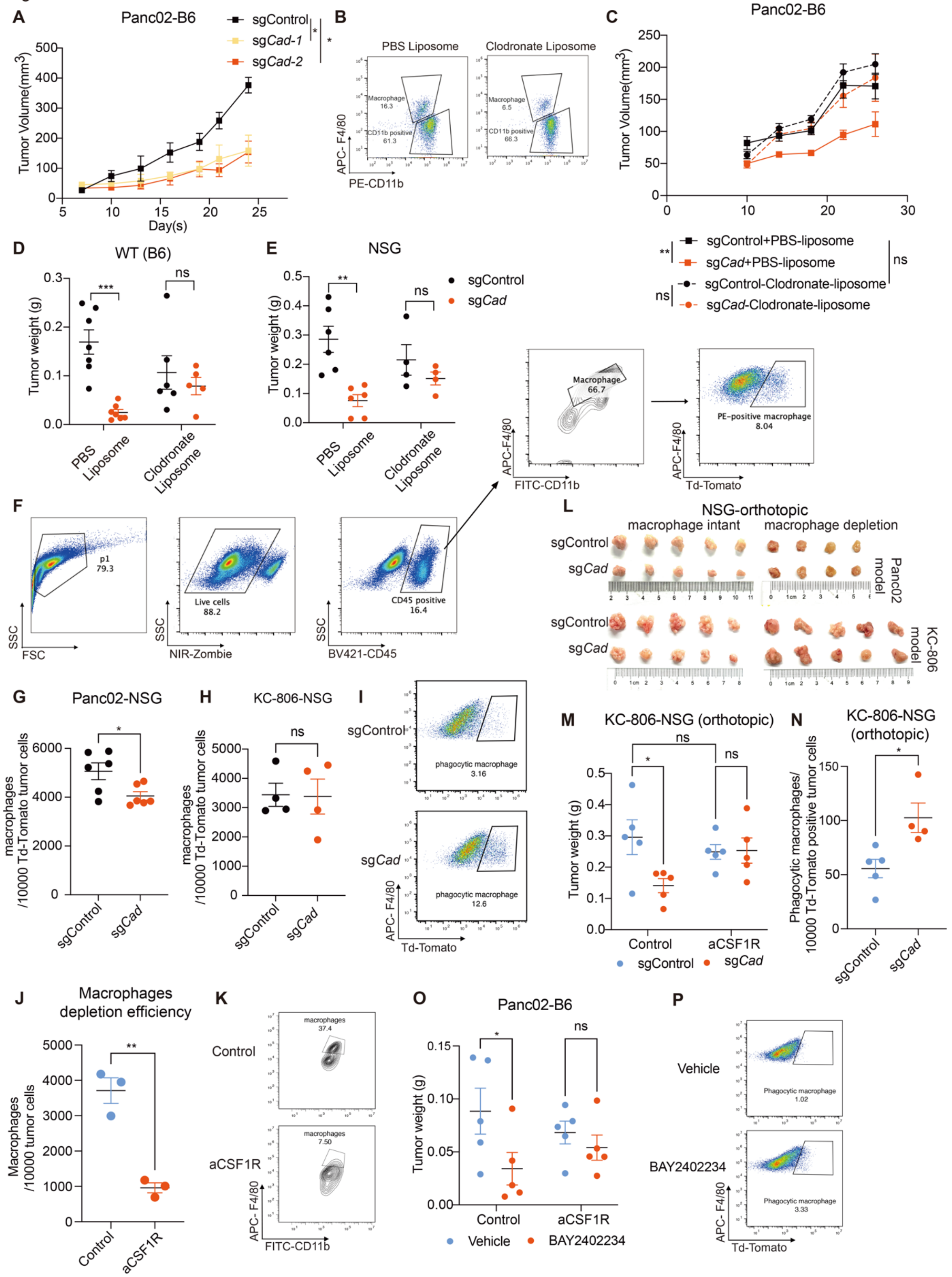
A
Panc02-B6
sgControl
sgCad-1
sgCad-2
Tumor Volume(mm3)
Day(s)
B
PBS Liposome
Clodronate Liposome
Macrophage 16.3
CD11b positive 61.3
Macrophage 6.5
CD11b positive 66.3
APC- F4/80
PE-CD11b
C
Panc02-B6
Tumor Volume(mm3)
sgControl+PBS-liposome
sgCad+PBS-liposome
sgControl-Clodronate-liposome
sgCad-Clodronate-liposome
D
WT (B6)
Tumor weight (g)
PBS Liposome
Clodronate Liposome
E
NSG
sgControl
sgCad
Tumor weight (g)
PBS Liposome
Clodronate Liposome
Macrophage 66.7
APC-F4/80
FITC-CD11b
PE-positive macrophage 8.04
APC-F4/80
Td-Tomato
F
p1 79.3
SSC
FSC
Live cells 88.2
SSC
NIR-Zombie
CD45 positive 16.4
SSC
BV421-CD45
L
NSG-orthotopic
macrophage intant
macrophage depletion
sgControl
sgCad
Panc02 model
sgControl
sgCad
KC-806 model
G
Panc02-NSG
macrophages /10000 Td-Tomato tumor cells
sgControl
sgCad
H
KC-806-NSG
macrophages /10000 Td-Tomato tumor cells
sgControl
sgCad
I
sgControl
phagocytic macrophage 3.16
sgCad
phagocytic macrophage 12.6
APC- F4/80
Td-Tomato
M
KC-806-NSG (orthotopic)
Tumor weight (g)
Control
aCSF1R
sgControl
sgCad
N
KC-806-NSG (orthotopic)
Phagocytic macrophages/ 10000 Td-Tomato positive tumor cells
sgControl
sgCad
J
Macrophages depletion efficiency
Macrophages /10000 tumor cells
Control
aCSF1R
K
Control
macrophages 37.4
aCSF1R
macrophages 7.50
APC- F4/80
FITC-CD11b
O
Panc02-B6
Tumor weight (g)
Control
aCSF1R
Vehicle
BAY2402234
P
Vehicle
Phagocytic macrophage 1.02
BAY2402234
Phagocytic macrophage 3.33
APC- F4/80
Td-Tomato

Figure S3. Inactivation of *de novo* pyrimidine synthesis pathway suppresses tumor growth *in vivo* and enhances macrophage-mediated phagocytosis, Related to Figure 2 and Figure 3.

(**A**) Growth curves of control or *Cad* KO Panc02 tumors in wild-type B6 mice.

(**B**) Representative FACS showing *in vivo* macrophage depletion efficiency by clodronate liposome. TAMs were gated on F4/80$^+$ and Cd11b$^+$.

(**C**) Growth curve of control or *Cad* KO Panc02 tumors following treatment of control vehicle (PBS liposome) or clodronate liposomes in wild-type B6 mice.

(**D-E**) Tumor weight of control or *Cad* KO Panc02 tumors following treatment of control vehicle (PBS liposome) or clodronate-liposome in wild-type B6 (**D**) mice or in NSG mice (**E**).

(**F**) Gating strategy of phagocytic macrophages, cells were determined based on double positive for F4/80 and Td-Tomato.

(**G-H**) *In vivo* quantification of the numbers of infiltrated macrophages in control and *Cad* KO Panc02 (**G**) and KC-806 (**H**) tumors, respectively.

(**I**) Representative FACS plots showing the percentage of phagocytic macrophages in KC-806-Td-Tomato tumors of Figure **2E**.

(**J-K**) Macrophage depletion efficiency. *In vivo* quantification of infiltrated macrophages in Panc02 orthotopic tumors treated with control vehicle or aCSF1R antibody (**J**). Representative flow plots were shown (**K**).

(**L**) Tumor images that are presented in Figures **3D** and **S3M**.

(**M**) $5x10^5$ of control or *Cad* KO KC-806 cells were orthotopically implanted into NSG mice, with or without aCSF1R antibody treatment. Tumor weight was measured on day 16 post-implantation.

(**N**) Quantification of normalized cell number of phagocytic macrophages in KC-806-Td-Tomato orthotopic tumors.

(**O**) Statistical analysis of tumor weight in Figure **3I**.

(**P**) Representative flow plots of Figure **3J**.

Data are represented as mean ± SEM and analyzed by mixed-effects model (REML) test (**A, C**), two-way ANOVA (**D-E, M, O**), or unpaired t-test (**G-H, J, N**). $^*P < 0.05$, $^{**}P < 0.01$ and $^{***}P < 0.001$. All data are representative of at least 2 independent experiments.

Figure S4

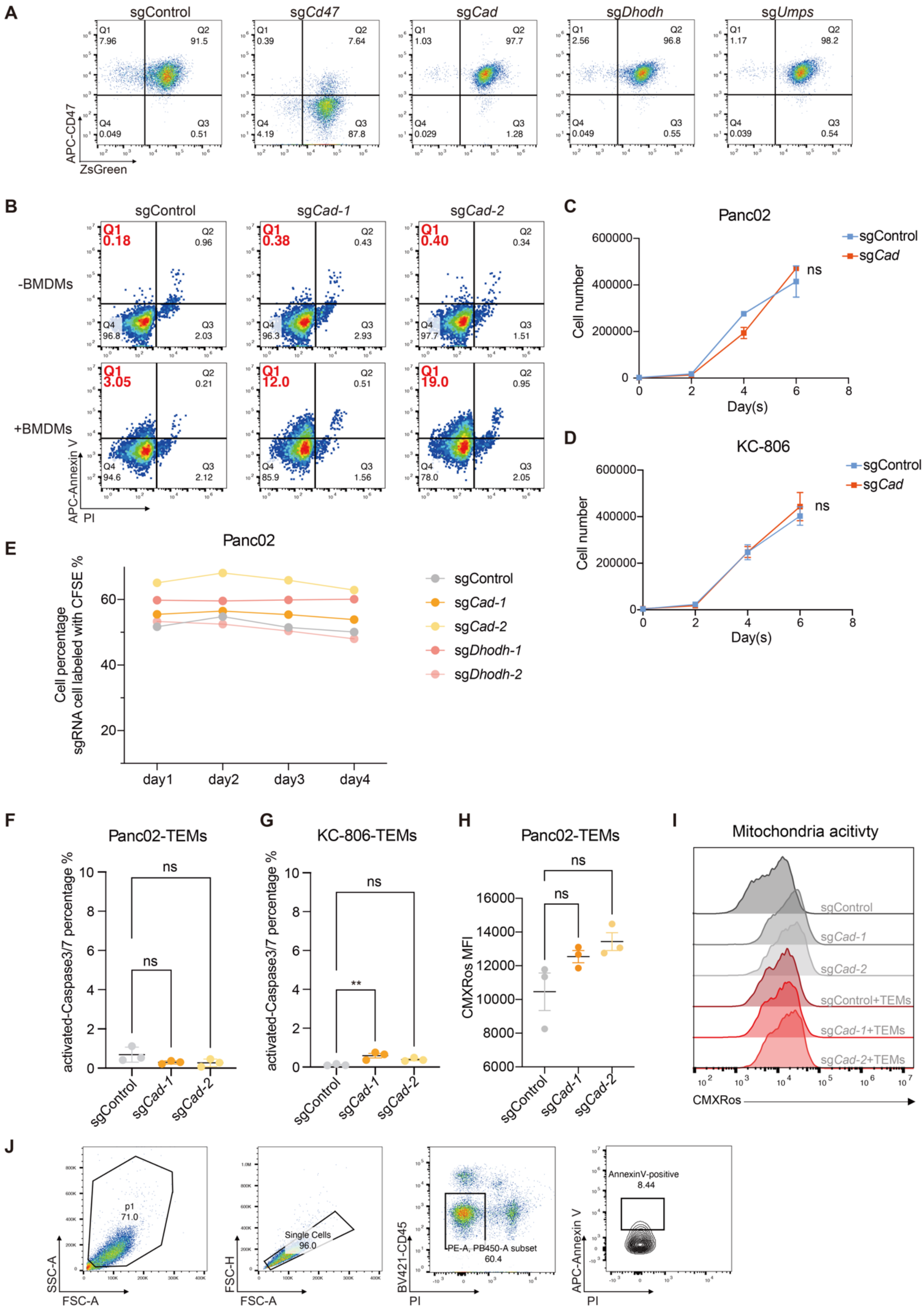
A
sgControl
sgCd47
sgCad
sgDhodh
sgUmps
APC-CD47
ZsGreen
B
sgControl
sgCad-1
sgCad-2
-BMDMs
+BMDMs
APC-Annexin V
PI
C
Panc02
sgControl
sgCad
ns
Cell number
Day(s)
D
KC-806
E
Panc02
Cell percentage
sgRNA cell labeled with CFSE %
day1
day2
day3
day4
sgDhodh-1
sgDhodh-2
F
Panc02-TEMs
activated-Caspase3/7 percentage %
G
KC-806-TEMs
H
CMXRos MFI
I
Mitochondria acitivty
sgControl+TEMs
sgCad-1+TEMs
sgCad-2+TEMs
CMXRos
J
SSC-A
FSC-A
FSC-H
BV421-CD45
Single Cells
AnnexinV-positive

Figure S4. Inactivation of *Cad* increased the exposure of phosphatidylserine but did not induce apoptosis in tumor cells in the presence of macrophages, Related to Figure 4.

(**A**) FACS analysis of CD47 expression level of Panc02-Fc cells in which indicated genes were knocked out by CRISPR/Cas9.

(**B**) Panc02 cells were either cultivated alone or co-cultured with BMDMs. The levels of Annexin V^+ PI^- cells were quantified by FACS. Representative FACS plots were shown. This panel represents the same experiment as shown in Figure **4A.**

(**C-D**) *In vitro* cell growth curves of control or *Cad* KO panc02(**C**) or KC-806(**D**) cells. Tumor cells were plated at 5000 cells per well on day 0, and cell numbers were counted every two days.

(**E**) Control or specific gene KO Panc02 cells were labeled with CFSE and mixed with sgControl Panc02 cells at a 1:1 ratio, the percentages of CFSE-positive cells were measured by FASC every day.

(**F-G**) The percentage of activated caspase3/7 in Annexin V^+ $Zombie^-$cell population. Control or *Cad* KO Panc02 (**F**) or KC-806 (**G**) cells were co-cultured with tumor-educated macrophages (TEMs) for 24 hours. Following co-culture, cells were stained with Annexin V, Zombie-NIR, and CellEvent Caspase-3/7 Green. The percentages of activated Caspase-$3/7^+$ cells within the Annexin V^+ Zombie- population are shown.

(**G-I**) Mitochondrion activity detected by Red-CMXRos. Control or *Cad* KO Panc02 (cells were co-cultured with tumor-educated macrophages (TEMs) for 24 hours. Following co-culture, cells were stained with Annexin V, Zombie-NIR, and Red-CMXRos. Statistics (**G**) and representative FACS plots (**I**) were shown.

(**J**) Gating strategy for annexin V staining *in vivo*. This panel represents the same experiment as shown in Figure **4H-I.**

For panels **C-D**, data are represented as mean ± SD, and analyzed by two-way ANOVA.

For panels **F-H**, data are represented as mean ± SD, and analyzed by one-way ANOVA. $^{**}P < 0.01$.

All data are representative of at least 2 independent experiments.

Figure S5

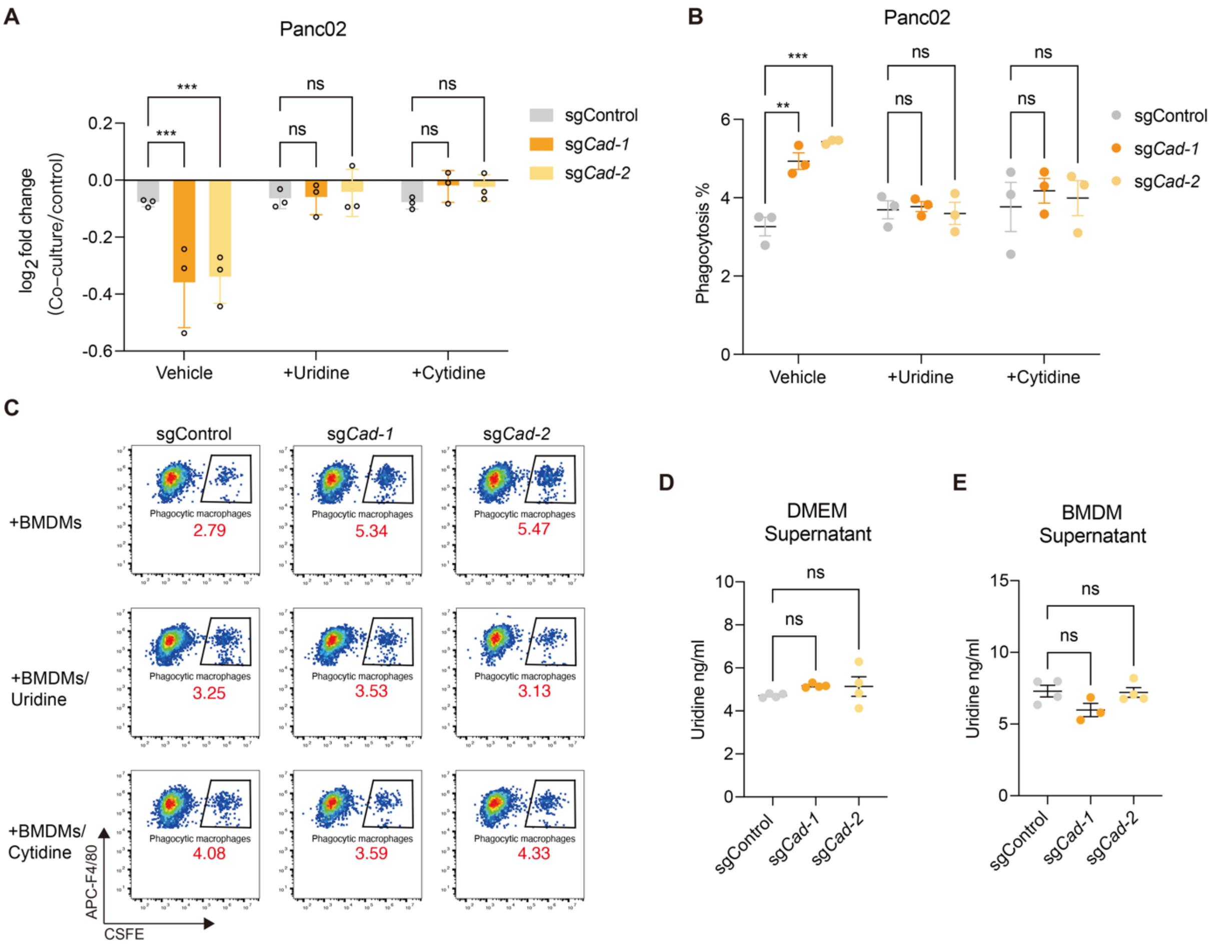

Figure S5. Macrophages suppress the salvage pathway of pyrimidine synthesis in tumor cells, Related to Figure 5.

(**A**) Tumor-macrophage co-culture experiments in the presence of indicated metabolites, including uridine (200µM) or cytidine (200µM), supplemented to the culture medium. Control Panc02 cells were mixed with CSFE-labelled cells transduced with sgRNA targeting indicated genes. The cell mixtures were then co-cultured with BMDMs. Log_2 fold changes of the percentage KO cells upon co-culture with BMDMs were presented.

(**B**) Phagocytosis assay of BMDMs with CFSE labeled Panc02 cells in the presence of indicated metabolites supplementation. The percentages of phagocytosis (F4/80$^+$ CSFE$^+$) were quantified by FACS, same experiments of Figure **5D**.

(**C**) Representative flow plots of Figure **5D** and Figure **S5B**.

(**D-E**) LC-MS analysis of indicated metabolites in cell culture supernatants derived from Panc02 cells transduced with indicated sgRNAs. Supernatants were harvested after culturing for 24 hours.

Data are represented as mean ± SD and analyzed by two-way ANOVA(**A-B**) or one-way ANOVA (**D-E**). $^{**}P < 0.01$ and $^{***}P < 0.001$. All data are representative of at least 2 independent experiments**.**

Figure S6

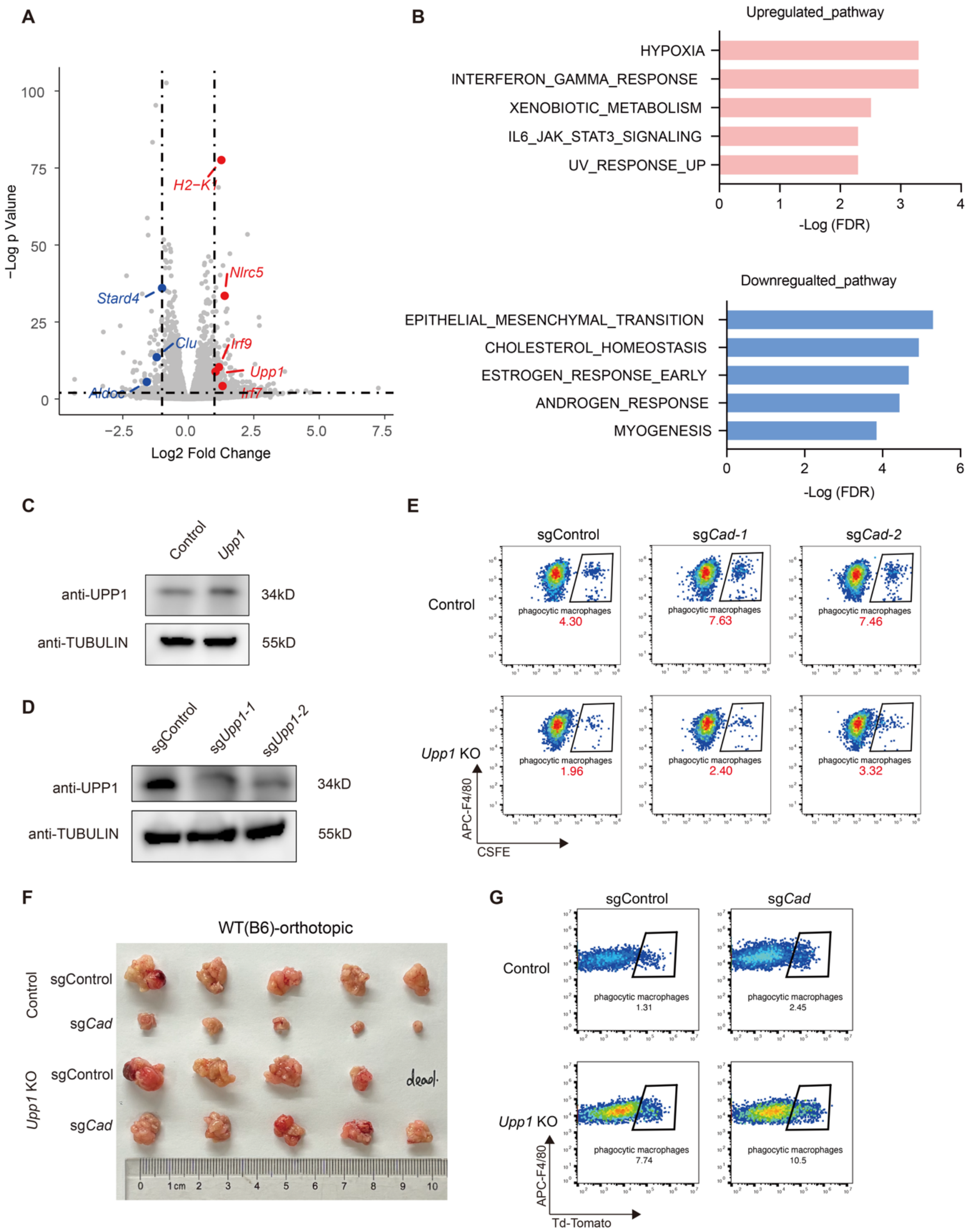

Figure S6. Macrophages affect the metabolic pathways in tumor cells, Related to Figure 6.

(**A**) Volcano plot of RNA-seq analysis showing the differentially expressed genes in Panc02 cells upon co-culture with BMDMs. *Upp1* and genes related to interferon signaling (upregulated) and cholesterol homeostasis (downregulated) were highlighted.
(**B**) GSEA (Gene Set Enrichment) analysis of RNA-seq analysis showing the upregulated (upper) and downregulated (bottom) pathways in *Cad* KO Panc02 cells upon co-culture with BMDMs for 24 hours.
(**C-D**) Western blot analysis of *Upp1* protein level in control, Panc02 cells with *Upp1* over-expression (**C**), or *Upp1* KO (**D**).
(**E**) Representative flow plots of Figure **6F**.
(**F**) Tumor images that are presented in Figure **6J**.
(**G**) Representative flow plots of Figure **6K**.

Data are representative of at least 2 independent experiments (**C-D**).

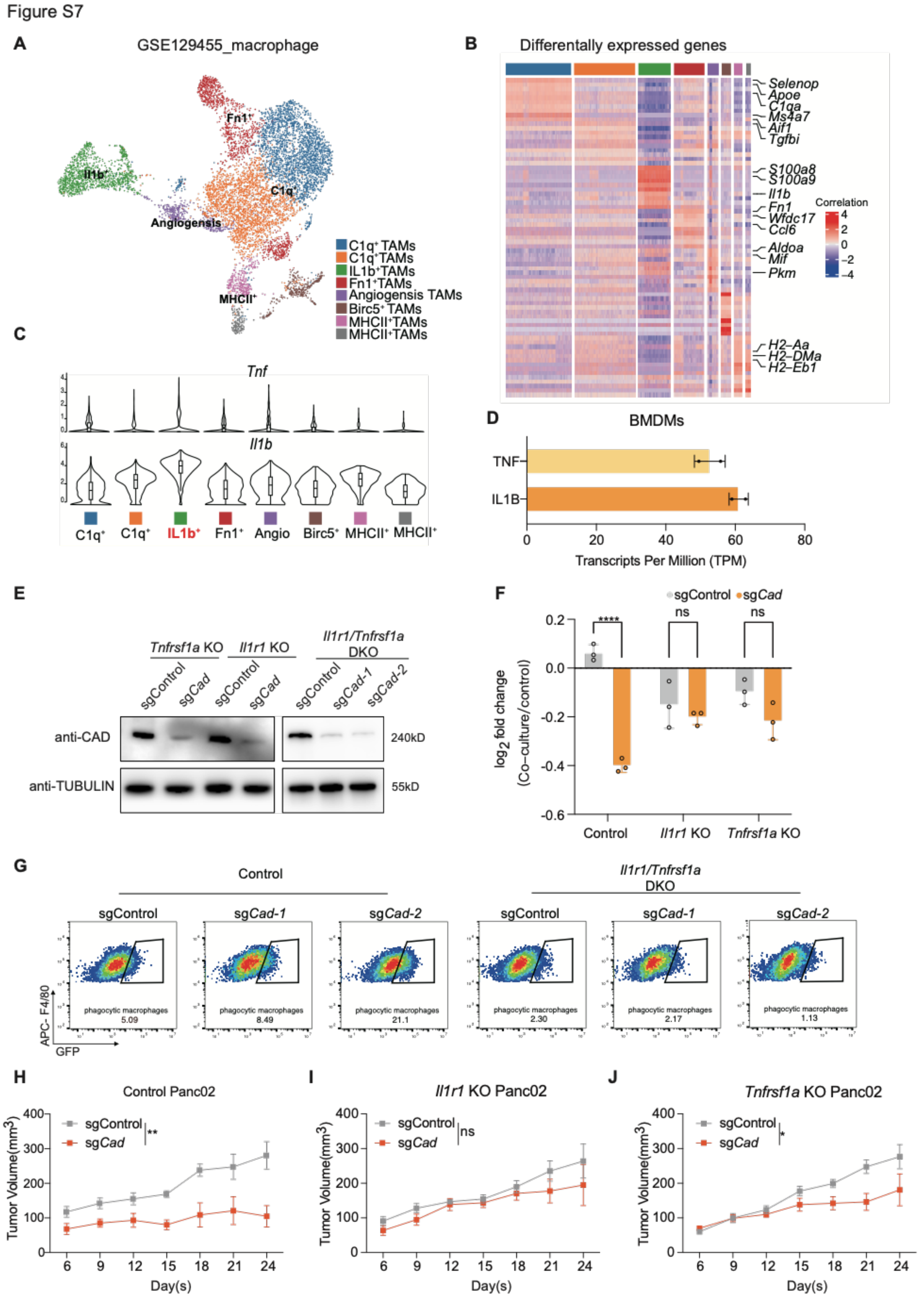

Figure S7. The roles of TNFa and IL-1 cytokines in regulating *Cad* KO related phenotype,

Related to Figure 7.

(**A-C**) Single-cell RNAseq analysis of the expression of IL1B and TNF in TAMs in a *K-ras/P53*-induced pancreatic tumor model. Analysis of TAMs using sc-RNAseq data generated from the $Kras^{G12D}$/*P53* KO-induced pancreatic cancer model (GSE129455). UMAP plot showing the distribution of different TAM subsets (**A**), annotated based on signature gene expression profiles presented in (**B**). Violin plots showing the expression levels of *Il1b* and *Tnf* across different TAM clusters (**C**).

(**D**) The expression of *Il1b* and *Tnf* in BMDMs. BMDMs were differentiated from monocytes for 7 days and then harvested for RNA-seq. Transcripts per million (TPM) of *Il1b* and *Tnf* are shown.

(**E**) Western blot analysis of *Cad* protein level to determine *Cad* KO efficiency in TNF receptor (*Tnfrsf1a*) KO, IL-1 receptor (*Il1r1*) KO, or double KO(*Il1r1/Tnfrsf1a*) Panc02 cells.

(F) Control, *Tnfrsf1a* KO, *Il1r1* KO Panc02 cells were transduced with control sgRNAs, or sgRNAs targeting *Cad*. These cells ($pHrodo^{+}$) were then mixed with control cells under the same genetic background (e.g. parental, *Tnfrsf1a* KO, *Il1r1* KO). The cell mixtures were then co-cultured with BMDMs for 24 hours for phagocytosis. Log_2 fold change of the percentage of $pHrodo^{+}$ cells upon co-culture with BMDMs was presented.

(**G**) Representative flow plots of Figure **7H**.

(H-J) Growth curves of control or *Cad* KO in parental Panc02 (**H**), *Il1r1* KO (**I**), or *Tnfrsf1a* KO (**J**) Panc02 tumors in NSG mice.

For panels **F**, data are represented as mean ± SD, and analyzed by two-way ANOVA.

For panels **H-J**, data are represented as mean ± SEM, and analyzed by mixed-effects model (REML) test.

$^{*}P < 0.05$, $^{**}P < 0.01$ and $^{****}P < 0.0001$.

Data are representative of at least 2 independent experiments (**E-J**).

Figure S8

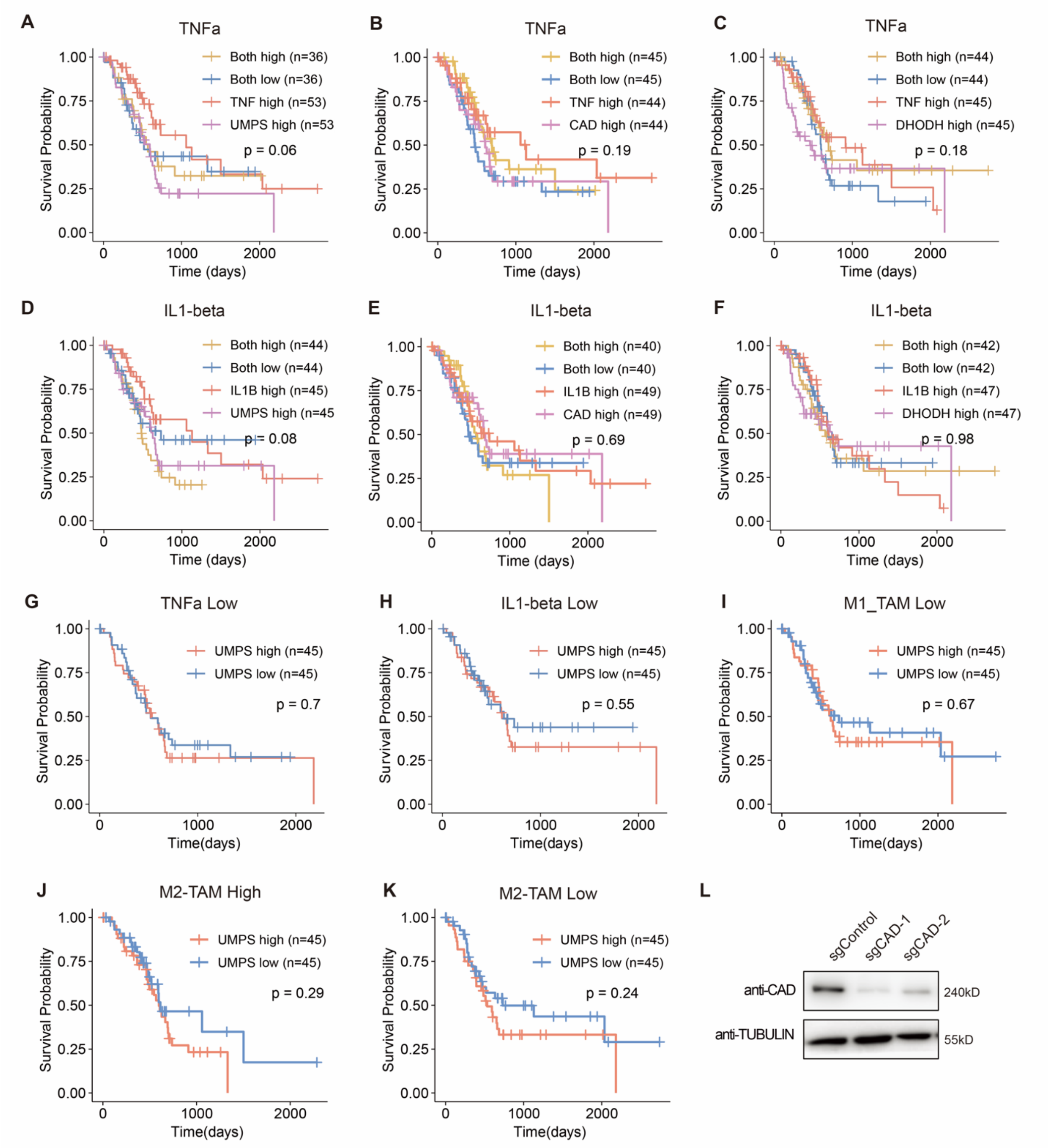

Figure S8. Human relevance for targeting *de novo* pyrimidine synthesis pathway in the presence of cytokine secrete macrophages, Related to Figure 8.

(**A-C**) Overall survival of TCGA PAAD patients based on the level of UMPS (**A**), CAD (**B**), DHODH (**C**) expression, and TNF.

(**D-F**) Overall survival of TCGA PAAD patients based on the level of UMPS (**D**), CAD (**E**), DHODH (**F**) expression, and IL1B.

(**G-I**) Overall survival of TCGA PAAD based on the lower expression of UMPS under the conditions of TNF expression level (**G**), IL1B expression level (**H**), or estimated level of M1-like macrophage infiltration (**I**).

(**J-K**) Overall survival of PAAD based on the level of UMPS expression under the conditions of estimated infiltration level of M2-like macrophage high (**J**) or low (**K**).

(**L**) Western blot analysis of knockout efficiency of CAD in PANC1 cells.

Statistical analyses were performed by using log-rank test. Data are representative of at least 2 independent experiments (**L**).

Supplementary Tables

Table S1. sgRNA sequences used in this study

TARGET	SEQUENCE (5'-3')
sg*Cad*-1	CTCAGAAACTCTGTTACGGG
sg*Cad*-2	CCGTGTGAGCCTACGCTACG
sg*Dhodh*-1	GGTATGGATTCAACAGCCAC
sg*Dhodh*-2	GTAGAAATGGTCGTCCCCCG
sg*Umps*-1	TCTGTCTGCCGATGTGTCGG
sg*Umps*-2	GATGTCATCATTGTAGGCCG
sg*Upp1*	GCTACGCCATGTATAAAGCC
sg*Il1r1*	GGATGATAAAGCCCCCGATG
sg*Tnfrsf1a*	GGGATATCGGCACATTAAAC
sgCAD-1	ACCTCCAGATATGGGAACCG
sgCAD-2	AAGTCAGTAACACACCATCG

Table S2. Summary of scRNA-seq dataset and cell number analyzed

Cancer type	Accession ID	Tumor_raw	Tumor_UPP1_retain	Macro_raw	Macro_TNF_retain	Macro_IL1B_retain
AEL	GSE142213	2492	42	590	0	93
ALL	GSE132509	21370	252	1412	85	511
ALL	GSE154109	5537	59	278	21	84
AML	GSE116256	12489	762	2893	111	197
AML	GSE154109	5384	765	2722	307	796
BRCA	EMTAB8107	7101	376	2508	824	1090
BRCA	GSE143423	4099	751	241	13	32
BRCA	GSE150660	1843	191	3161	136	370
CHOL	GSE138709	13464	5543	4370	721	2704
CHOL	GSE142784	750	156	435	8	2
CLL	GSE132065	36040	270	331	32	64
COAD	GSE146771	1040	458	1349	573	919

CRC	EMTAB8107	5497	1550	3296	372	1207
GBM	GSE131928	6648	2310	4809	530	2325
GBM	GSE138794	11846	1567	1262	335	704
GBM	GSE139448	12649	1522	602	128	140
GBM	GSE141982	4660	92	504	192	180
GBM	GSE84465	64	27	1843	560	936
HNSC	GSE103322	2488	1390	88	24	36
LSCC	GSE150321	6172	1651	664	357	421
MM	GSE117156	15839	181	472	14	9
NSCLC	EMTAB6149	8120	2392	18084	1785	3951
NSCLC	GSE117570	3367	1556	3203	434	1655
NSCLC	GSE127465	3995	724	7032	641	3232
NSCLC	GSE143423	9237	6792	2020	166	525
NSCLC	GSE150660	322	206	3005	88	61
OV	EMTAB8107	8887	1003	5692	443	1646
OV	GSE130000	8876	2212	259	48	104
OV	GSE154600	2320	810	8333	722	3468
PAAD	CRA001160	11401	4636	3327	205	636
PAAD	GSE111672	1489	208	210	7	54
PAAD	GSE141017	2462	1284	612	41	174
PAAD	GSE154778	10973	3291	1578	344	774
SKCM	GSE72056	1365	941	221	66	99
UVM	GSE139829	79105	13492	5663	2166	3226
