# Supplementary material for: Targeting plasticity in the pyrimidine synthesis pathway potentiates macrophage-mediated phagocytosis in pancreatic cancer models
Source: J Clin Invest. 2025 Nov 17;135(22):e193370. doi: 10.1172/JCI193370 (PMC12618067; doi:10.1172/JCI193370)
Supplement: Unedited blot and gel images [file jci-135-193370-s216.pdf]

Figure S2C

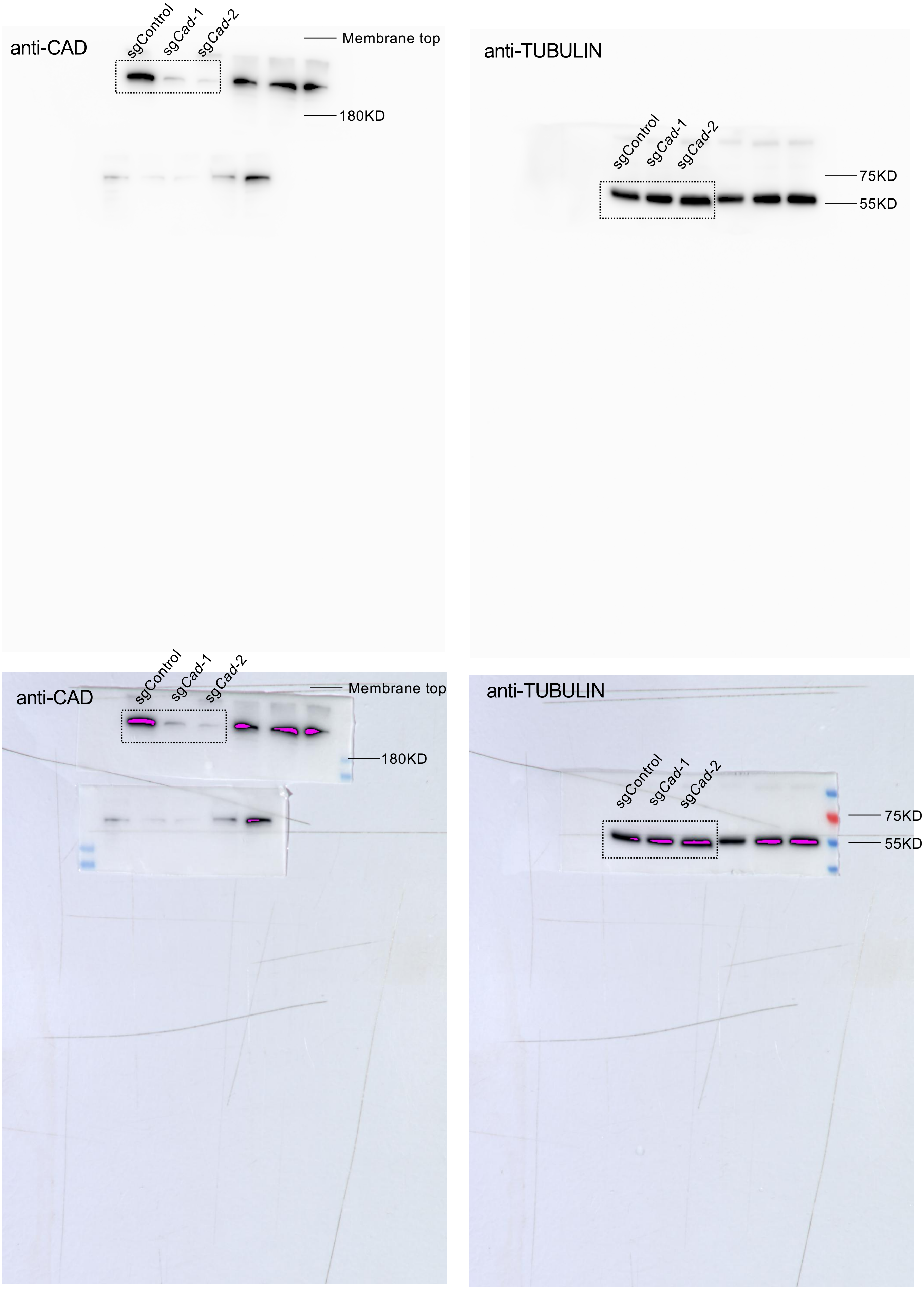
anti-CAD
sgControl
sgCad-1
sgCad-2
Membrane top
180KD
anti-TUBULIN
sgControl
sgCad-1
sgCad-2
75KD
55KD
anti-CAD
sgControl
sgCad-1
sgCad-2
Membrane top
180KD
anti-TUBULIN
sgControl
sgCad-1
sgCad-2
75KD
55KD

Figure S2D

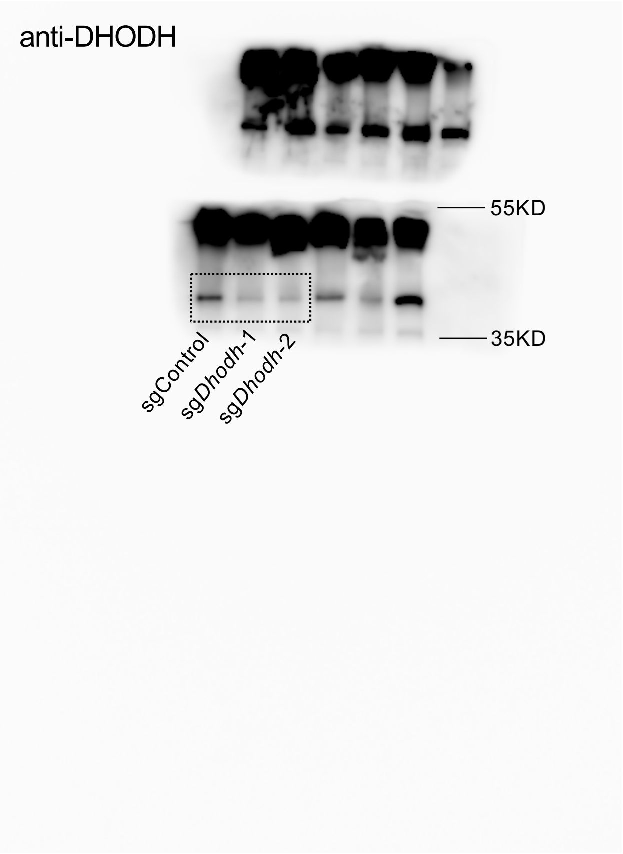
anti-DHODH
55KD
35KD
sgControl
sgDhodh-1
sgDhodh-2

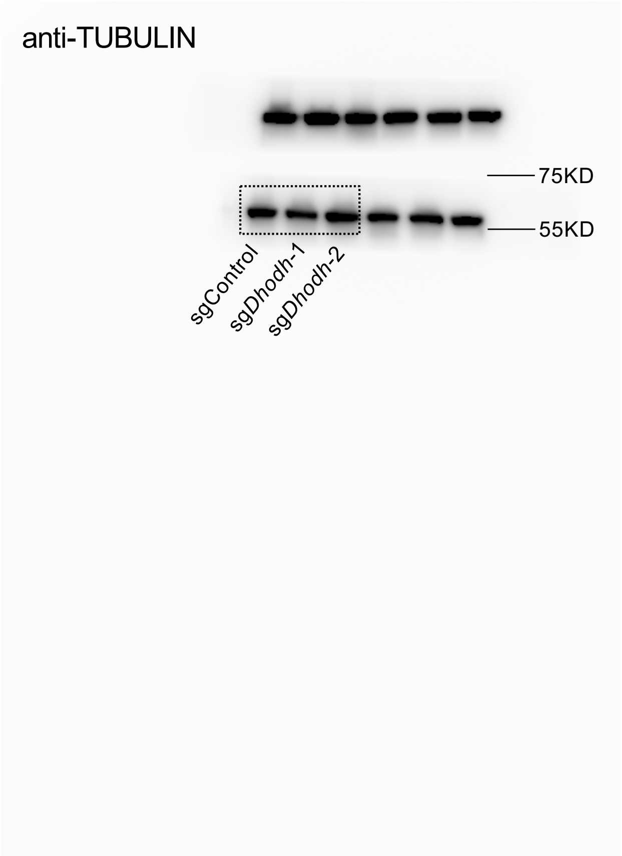
anti-TUBULIN
75KD
55KD
sgControl
sgDhodh-1
sgDhodh-2

Figure S2E

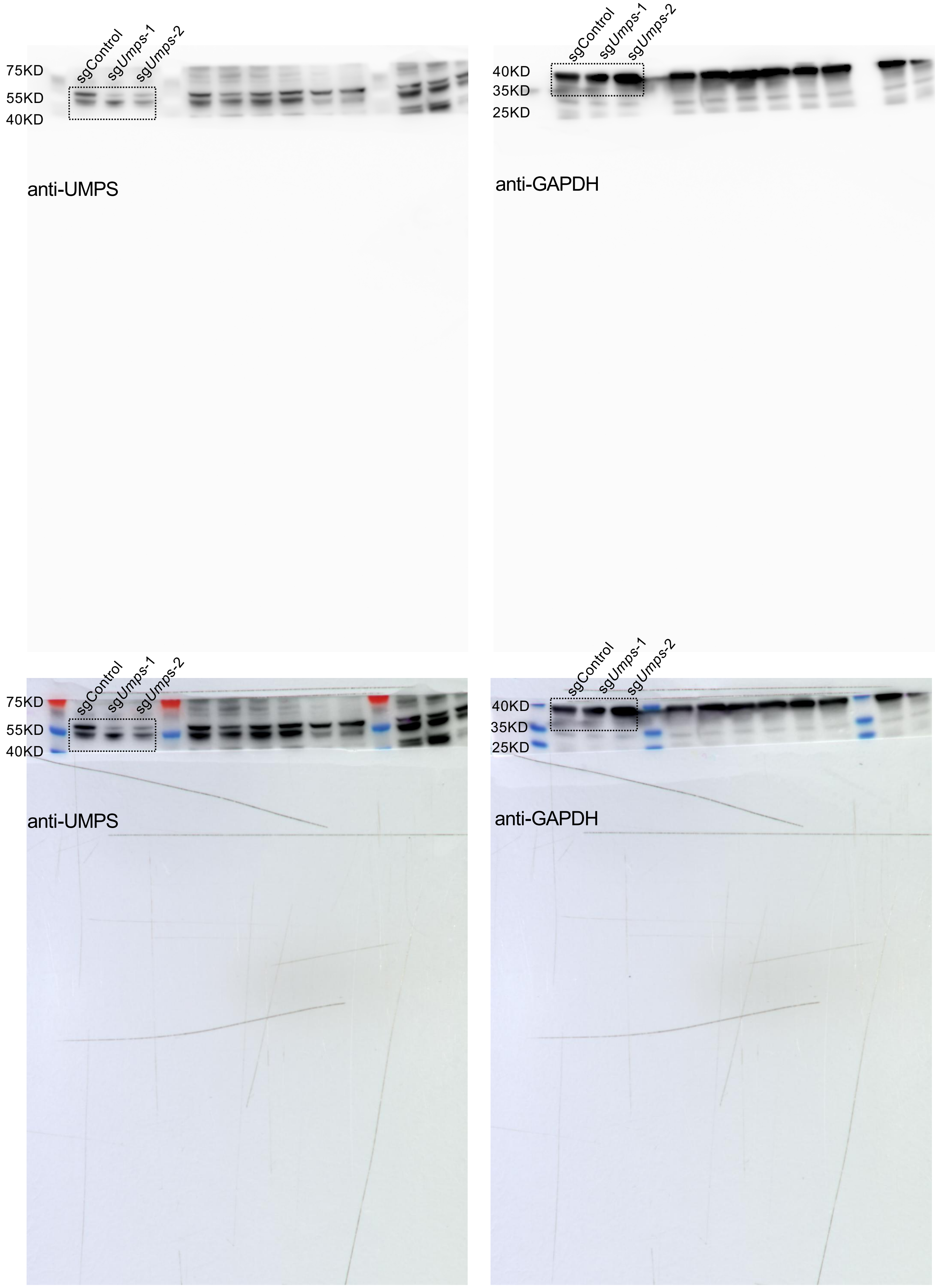
sgControl
sgUmps-1
sgUmps-2
75KD
55KD
40KD
anti-UMPS
sgControl
sgUmps-1
sgUmps-2
40KD
35KD
25KD
anti-GAPDH
sgControl
sgUmps-1
sgUmps-2
75KD
55KD
40KD
anti-UMPS
sgControl
sgUmps-1
sgUmps-2
40KD
35KD
25KD
anti-GAPDH

Figure S6C

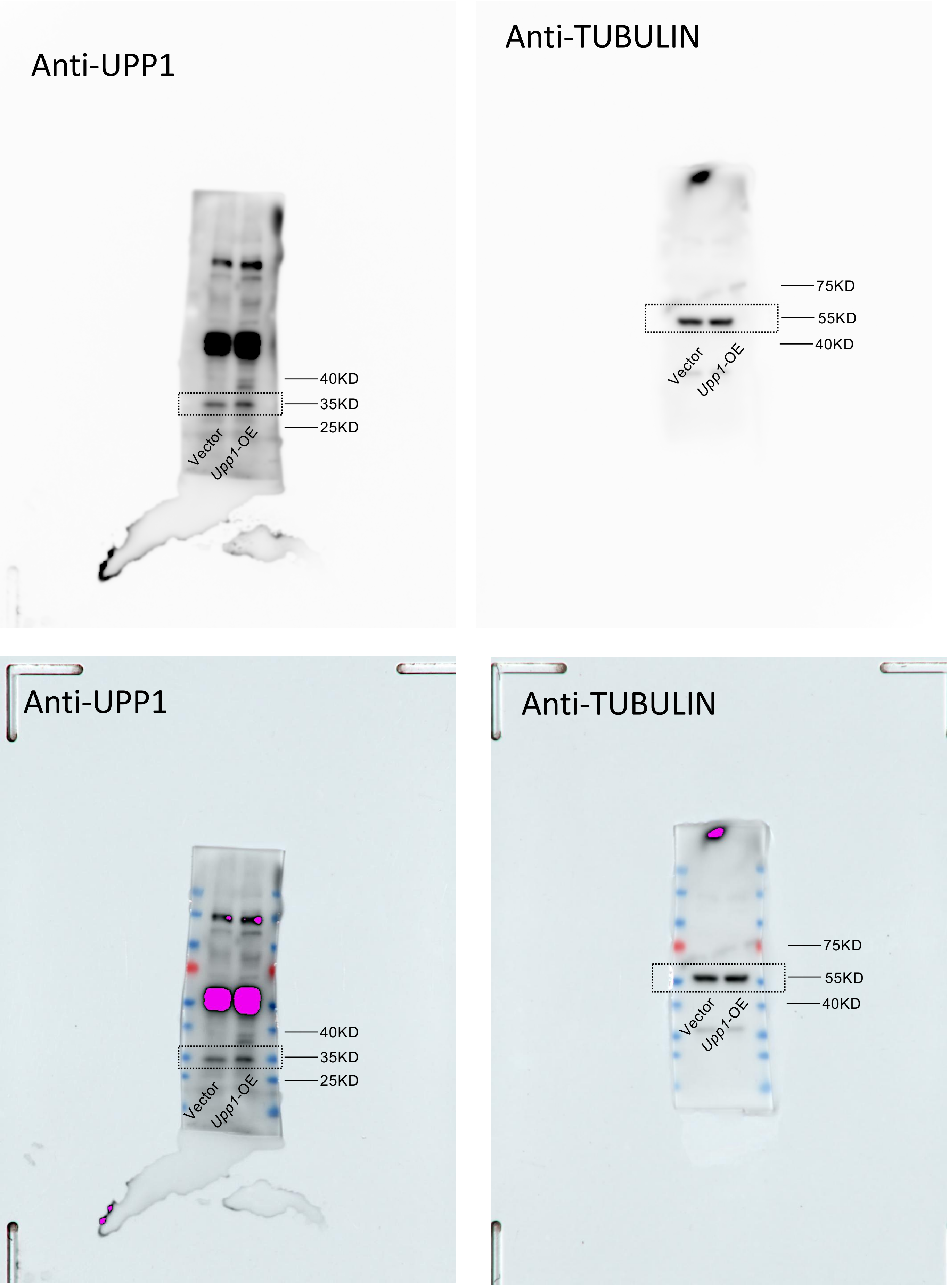

Anti-UPP1
40KD
35KD
25KD
Vector
Upp1-OE
Anti-TUBULIN
75KD
55KD
40KD
Vector
Upp1-OE
Anti-UPP1
40KD
35KD
25KD
Vector
Upp1-OE
Anti-TUBULIN
75KD
55KD
40KD
Vector
Upp1-OE

Figure S6D

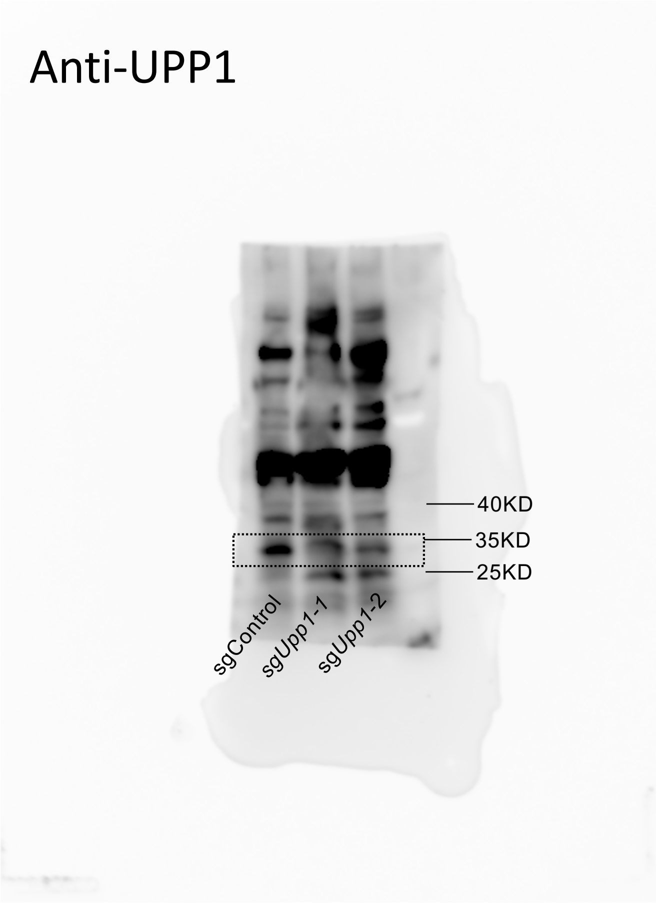
Anti-UPP1
40KD
35KD
25KD
sgControl
sgUpp1-1
sgUpp1-2

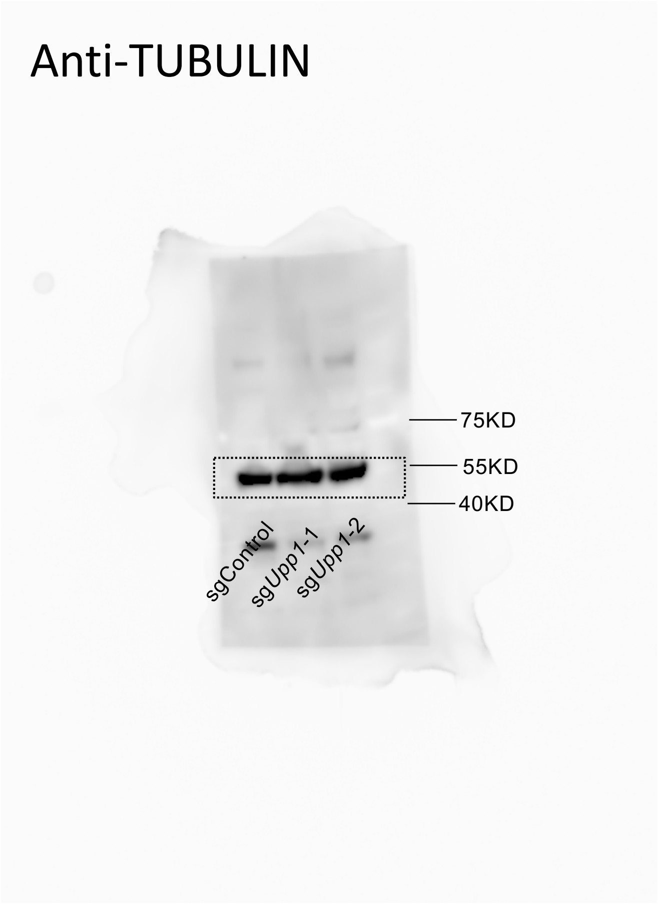
Anti-TUBULIN
75KD
55KD
40KD
sgControl
sgUpp1-1
sgUpp1-2

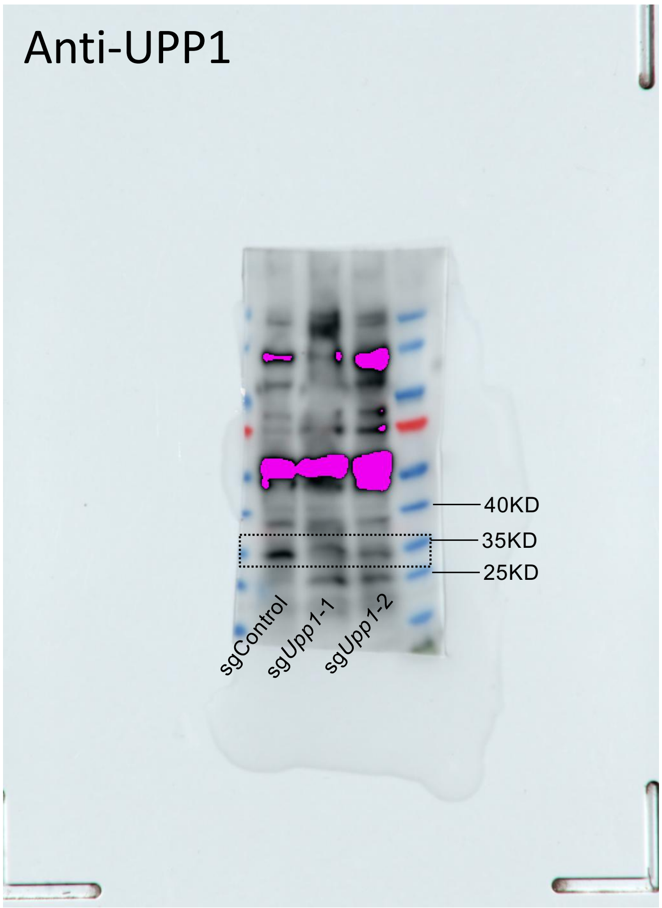
Anti-UPP1
40KD
35KD
25KD
sgControl
sgUpp1-1
sgUpp1-2

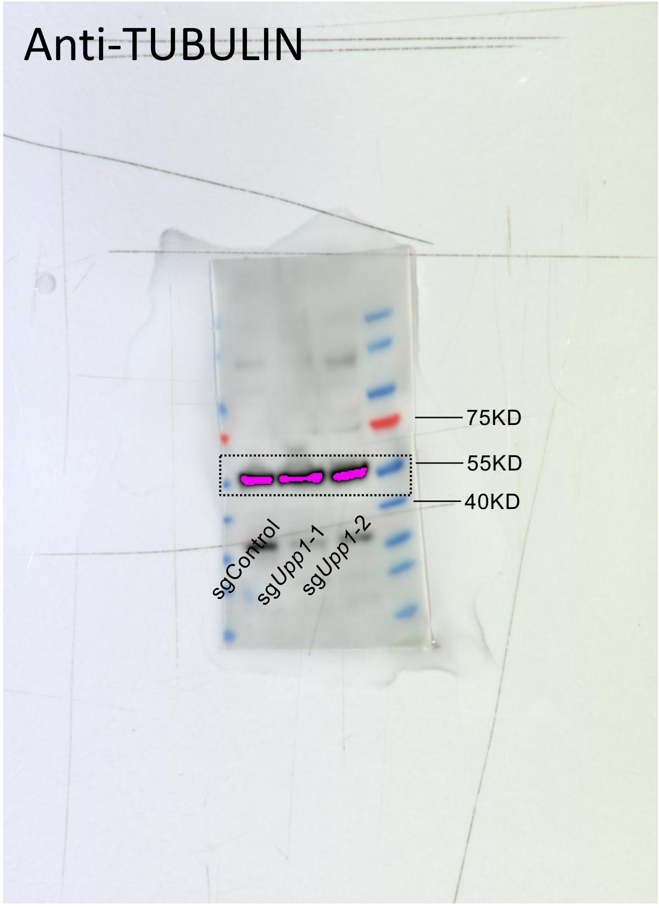
Anti-TUBULIN
75KD
55KD
40KD
sgControl
sgUpp1-1
sgUpp1-2

Figure S7E

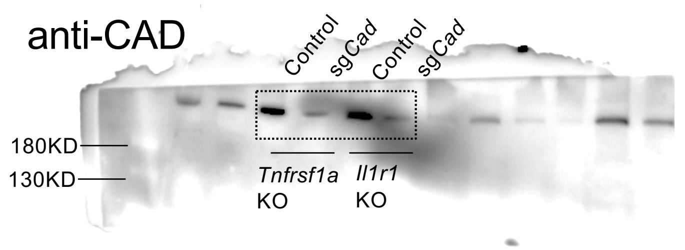
anti-CAD
Control
sgCad
Control
sgCad
180KD
130KD
Tnfrsf1a KO
Il1r1 KO

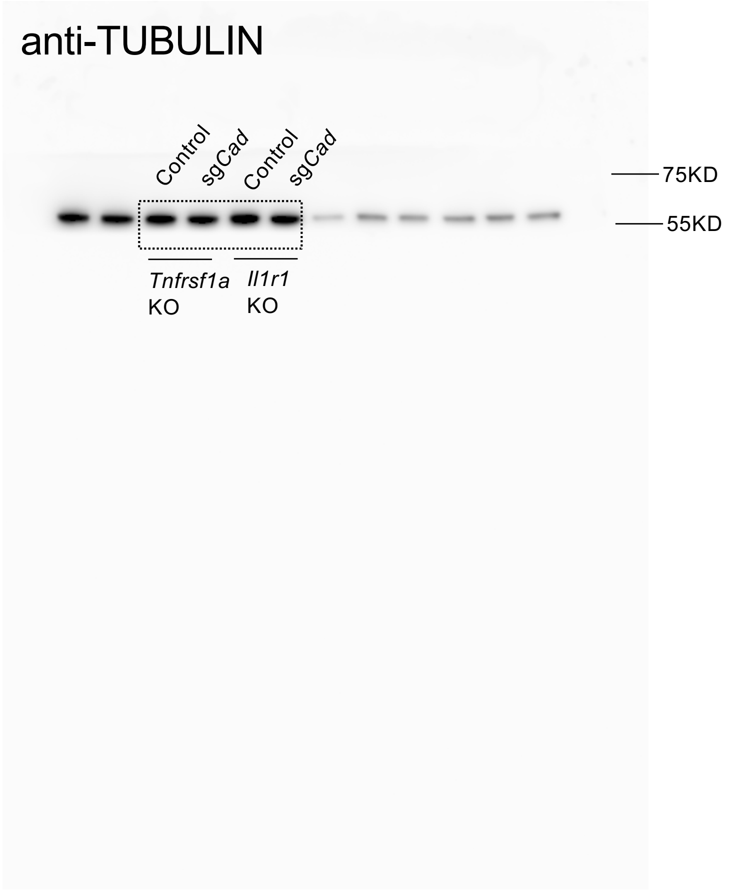
anti-TUBULIN
Control
sgCad
Control
sgCad
75KD
55KD
Tnfrsf1a KO
Il1r1 KO

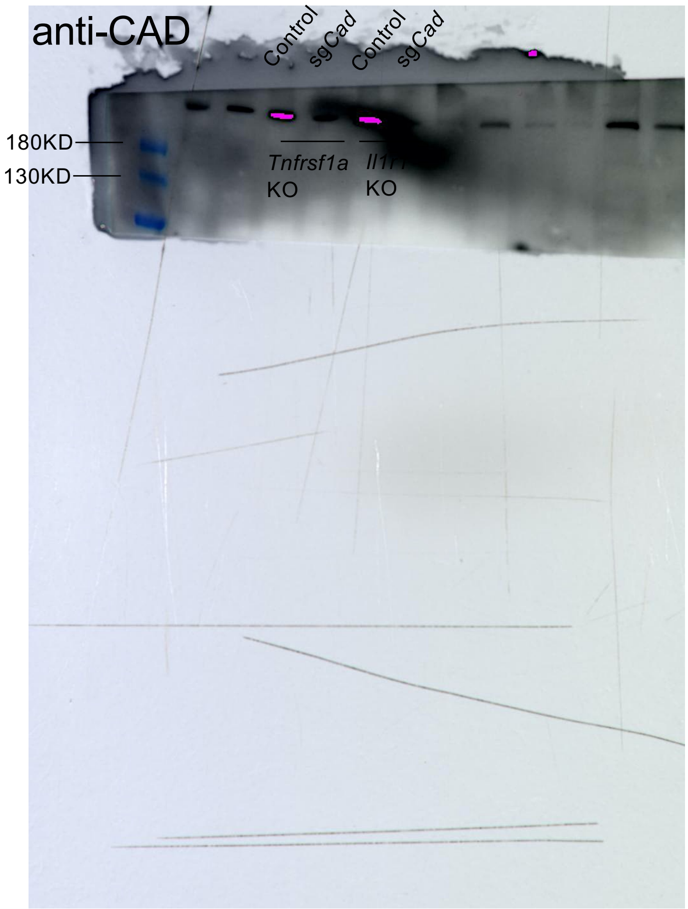
anti-CAD
Control
sgCad
Control
sgCad
180KD
130KD
Tnfrsf1a KO
KO

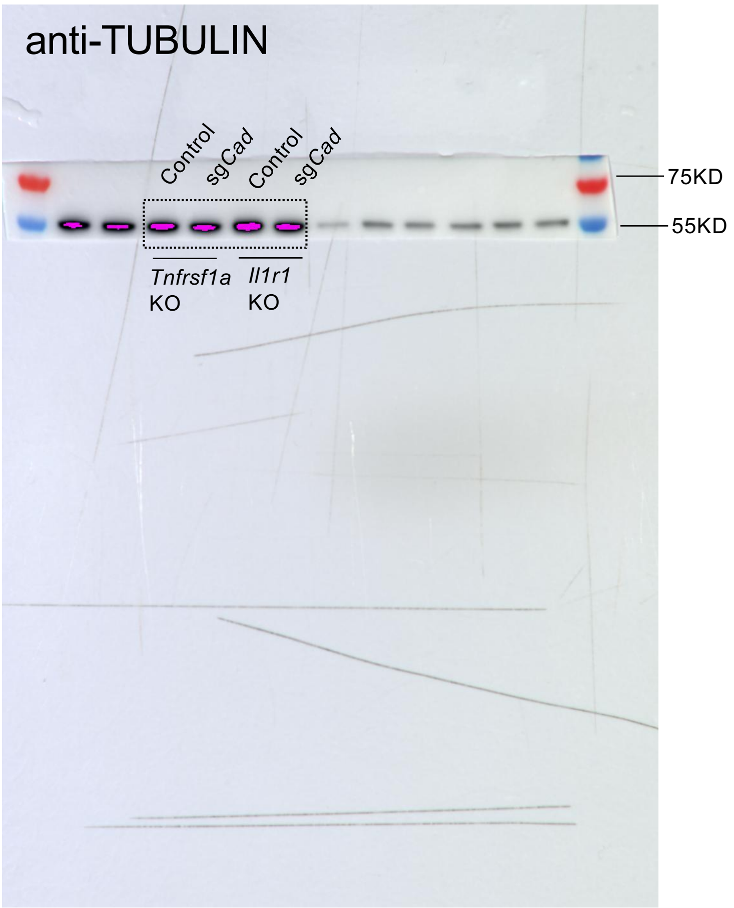
anti-TUBULIN
Control
sgCad
Control
sgCad
75KD
55KD
Tnfrsf1a KO
Il1r1 KO

Figure S7E

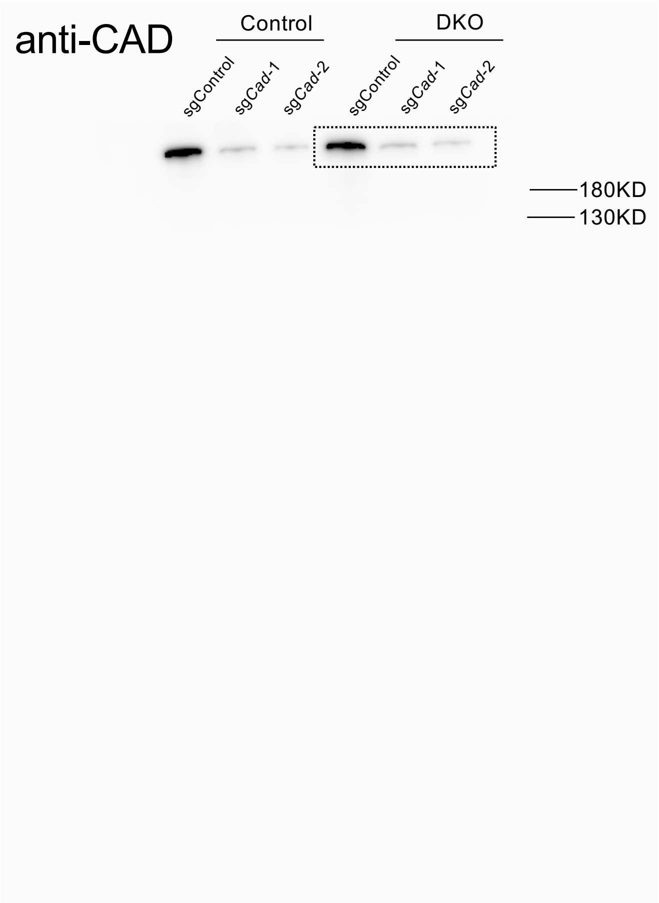
anti-CAD
Control
DKO
sgControl
sgCad-1
sgCad-2
sgControl
sgCad-1
sgCad-2
180KD
130KD

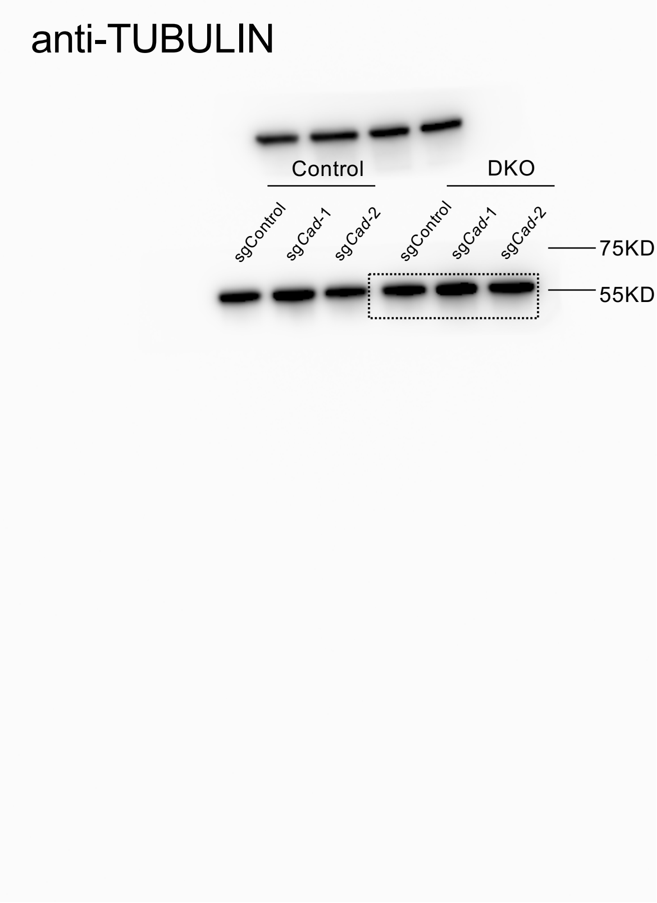
anti-TUBULIN
Control
DKO
sgControl
sgCad-1
sgCad-2
sgControl
sgCad-1
sgCad-2
75KD
55KD

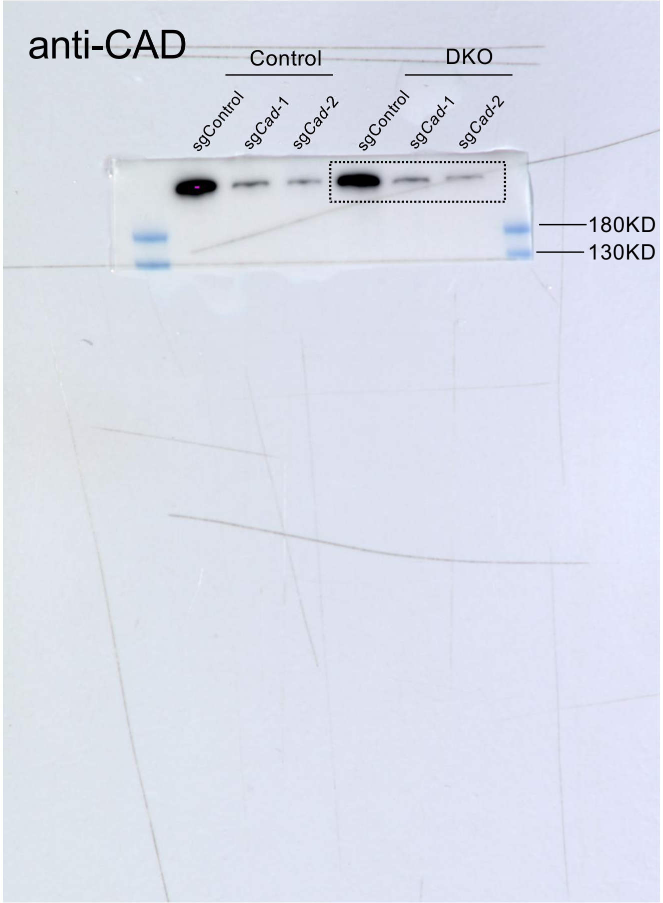
anti-CAD
Control
DKO
sgControl
sgCad-1
sgCad-2
sgControl
sgCad-1
sgCad-2
180KD
130KD

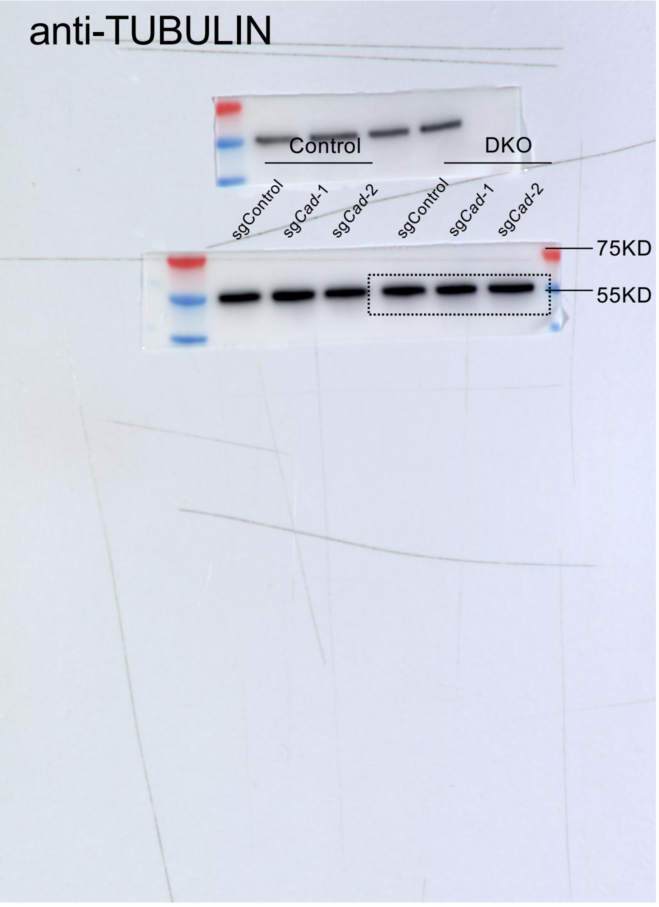
anti-TUBULIN
Control
DKO
sgControl
sgCad-1
sgCad-2
sgControl
sgCad-1
sgCad-2
75KD
55KD

Figure S8L

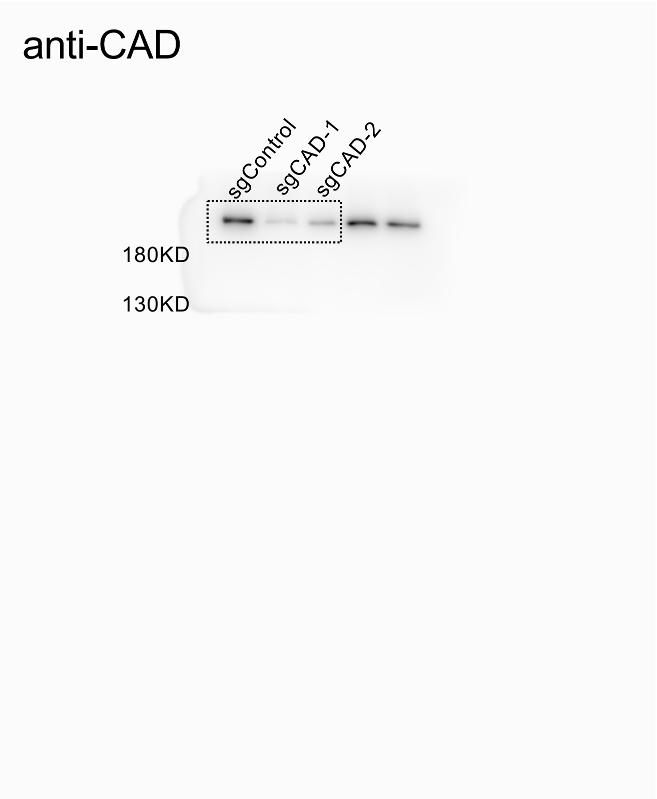
anti-CAD
sgControl
sgCAD-1
sgCAD-2
180KD
130KD

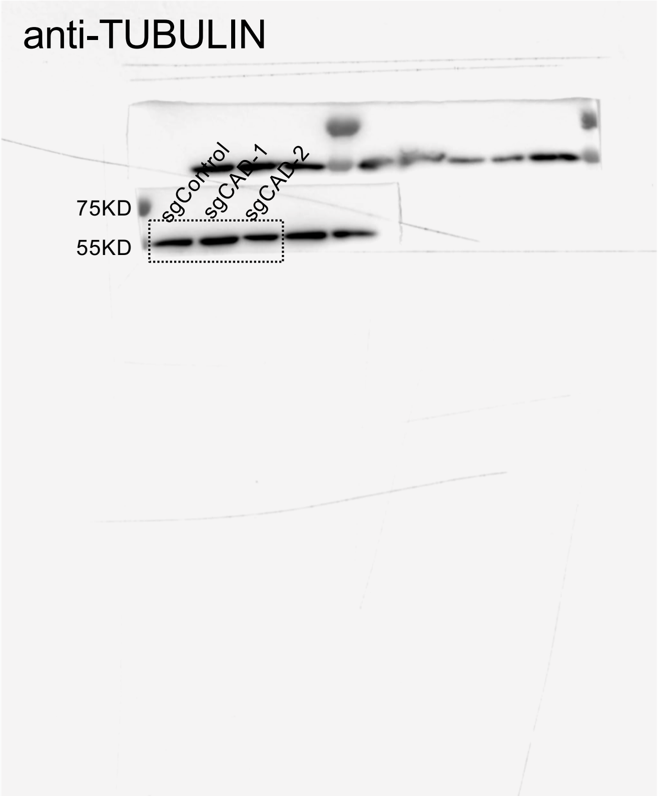
anti-TUBULIN
sgControl
sgCAD-1
sgCAD-2
75KD
55KD

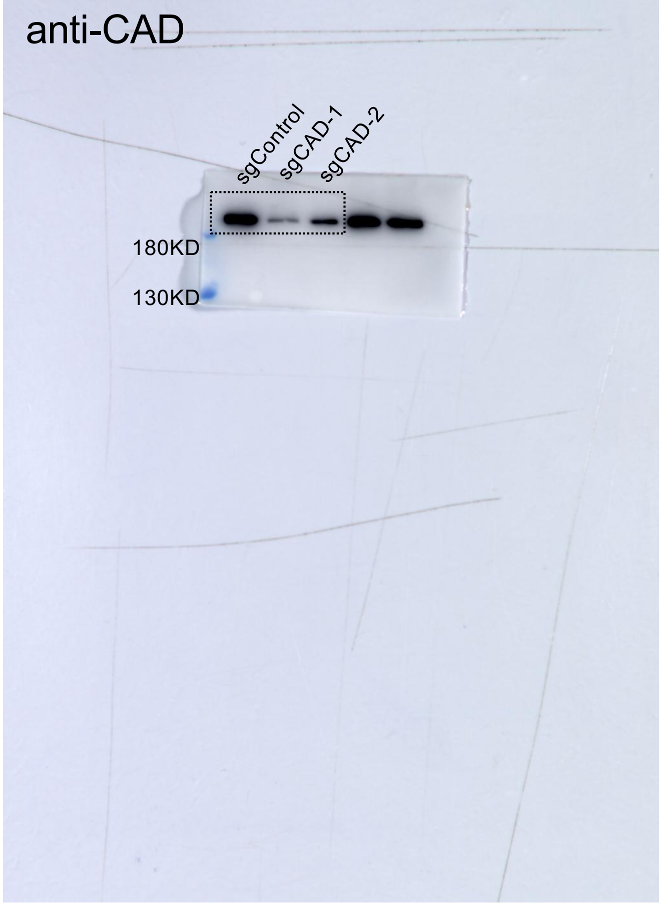
anti-CAD
sgControl
sgCAD-1
sgCAD-2
180KD
130KD

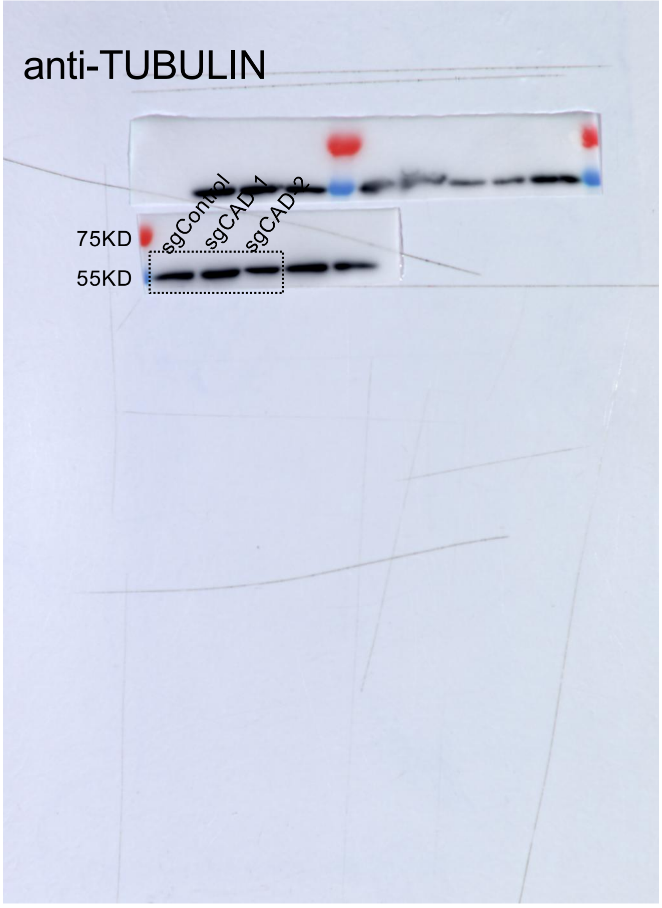
anti-TUBULIN
sgControl
sgCAD-1
sgCAD-2
75KD
55KD
